# Assessing the potential impacts of a revised set of on-farm nutrient and sediment ‘basic’ control measures for reducing agricultural diffuse pollution across England

**DOI:** 10.1016/j.scitotenv.2017.10.078

**Published:** 2018-04-15

**Authors:** A.L. Collins, J.P. Newell Price, Y. Zhang, R. Gooday, P.S. Naden, D. Skirvin

**Affiliations:** a Sustainable Agriculture Sciences Department, Rothamsted Research, North Wyke, Okehampton, Devon EX20 2SB, UK; b ADAS, Gleadthorpe, Meden Vale, Mansfield, Nottinghamshire NG20 9PD, UK; c ADAS, Titan 1 Offices, Coxwell Avenue, Wolverhampton Science Park, Wolverhampton WV10 9RT, UK; d CEH Wallingford, Maclean Building, Benson Lane, Crowmarsh Gifford, Wallingford, Oxfordshire OX10 8BB, UK

**Keywords:** Control measures, Costs, Efficacy, Nutrients, Sediment, Water Framework Directive

## Abstract

The need for improved abatement of agricultural diffuse water pollution represents cause for concern throughout the world. A critical aspect in the design of on-farm intervention programmes concerns the potential technical cost-effectiveness of packages of control measures. The European Union (EU) Water Framework Directive (WFD) calls for Programmes of Measures (PoMs) to protect freshwater environments and these comprise ‘basic’ (mandatory) and ‘supplementary’ (incentivised) options. Recent work has used measure review, elicitation of stakeholder attitudes and a process-based modelling framework to identify a new alternative set of ‘basic’ agricultural sector control measures for nutrient and sediment abatement across England. Following an initial scientific review of 708 measures, 90 were identified for further consideration at an industry workshop and 63 had industry support. Optimisation modelling was undertaken to identify a shortlist of measures using the Demonstration Test Catchments as sentinel agricultural landscapes. Optimisation selected 12 measures relevant to livestock or arable systems. Model simulations of 95% implementation of these 12 candidate ‘basic’ measures, in addition to business-as-usual, suggested reductions in the national agricultural nitrate load of 2.5%, whilst corresponding reductions in phosphorus and sediment were 11.9% and 5.6%, respectively. The total cost of applying the candidate ‘basic’ measures across the whole of England was estimated to be £450 million per annum, which is equivalent to £52 per hectare of agricultural land. This work contributed to a public consultation in 2016.

## Highlights

• Stakeholders scored ninety measures for water pollution from agriculture.
• Model optimisation shortlisted twelve measures for livestock and arable farms.
• Shortlisted measures reduced national nitrate load to rivers by 2.5%, sediment 5.6%.
• Shortlisted measures reduced national phosphorus load to rivers by 11.9%.
• Annual cost to farms at national scale was £450 M equating to £52 per hectare.

## Graphical abstract

**Figure:**
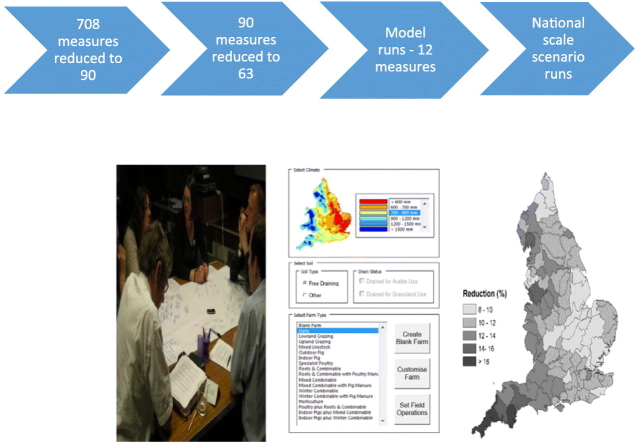

## 1 Introduction

Agricultural diffuse sources of pollution are recognised as the principal polluters of many rivers and lakes worldwide including those in the Baltic (Elofsson et al., 2003), Mediterranean (Panagopoulos et al., 2011), north America (Kramer et al., 2006), Europe (Lacroix et al., 2005, Crossman et al., 2013, Berger et al., 2017, Fischer et al., 2017, Mockler et al., 2017), Australia (Kroon, 2009, Thorburn, 2013, van Grieken et al., 2013, McDowell and Nash, 2013) and New Zealand (McDowell and Nash, 2013). The significant challenges posed by delivering effective control of diffuse pollution sources, including agriculture, mean that globally, the degradation of freshwater ecosystems has outpaced remedial action (e.g. Doole et al., 2013). In addition, climate change, land use change, and the need to provide food, water and other ecosystem services for a growing population have combined to create a ‘perfect storm’ (Beddington, 2009).

Since its introduction in 2000, the European Union (EU) Water Framework Directive (WFD) (Directive 222/60/EC; European Commission (EC), 2000, European Commission, 2012) has focussed much scientific research and policy team work across Member States on the problems of controlling diffuse agricultural pollution and especially those posed by elevated losses of nutrients and sediment. The EU WFD integrates economic analysis into water policy for governing environmental management and Annex III explicitly calls for analysis of costs and effectiveness to support the design of Programmes of Measures (PoMs) to help achieve ‘good ecological status’ (WATECO, 2003, Baylis et al., 2008, Balana et al., 2011, Ghebremichael et al., 2013, van Grieken et al., 2013, Balana et al., 2015).

Much of the scientific research driven by the EU WFD is focussed on improving the evidence base on the cost-effectiveness of specific pollution control measures at field scale (Deasy et al., 2009, Bailey et al., 2013, Destandau et al., 2013, Ockenden et al., 2012, Ockenden et al., 2014, Duffy et al., 2016, Vinten et al., 2017). However, whilst research work delivers fundamental experimental evidence on the costs and effectiveness of individual control measures in specific settings, environmental status is ultimately assessed at coarser scale (e.g. water body scale) meaning that evidence is increasingly required on the scope for combined or integrated diffuse agricultural pollution control measures to help achieve policy targets (Bouraoui and Grizzetti, 2014). Ongoing programmes are designed to deliver such evidence, including the Demonstration Test Catchments (DTC) initiative in England (McGonigle et al., 2012, McGonigle et al., 2014, Outram et al., 2014) and many other studies both in the EU and elsewhere (Gren et al., 1997, Elofsson, 2003, Berbel et al., 2011, Panagopoulos et al., 2011, Gren et al., 2013, Lescot et al., 2013, Panagopoulos et al., 2014, Roebeling et al., 2014, Rocha et al., 2015). The complexities of pollution mobilisation, transfer and delivery through river catchments mean, however, that monitored outcomes will take years to decades to confirm successful impacts arising from targeted on-farm remediation (Kronvang et al., 2005, Meals et al., 2010, Collins et al., 2014, McGonigle et al., 2014, Wang et al., 2016).

Given the need to inform policy in the short-term, a range of approaches has been used to perform analyses of the technically feasible costs and effectiveness of packages of pollution control measures including nonlinear (Brady, 2003) or linear mathematical programming (Azzaino et al., 2002, Froschl et al., 2008, Cardenas et al., 2011), process-based (including spatially-distributed) modelling of nutrient exports (Lam et al., 2010, Rocha et al., 2015) or critical source areas (Roebeling et al., 2009, Shang et al., 2012, Lescot et al., 2013, Chen et al., 2014, Roebeling et al., 2014, Perez-Martin et al., 2016, Teshager et al., 2017), hydro-economic (Yang et al., 2007) or bio-economic modelling (Schou et al., 2000, Semaan et al., 2007, Eory et al., 2013, Ferrant et al., 2013), agricultural sector programming (Ribaudo et al., 2001), abatement-cost curves using computable general equilibrium or partial equilibrium models (Moran et al., 2010, Doole, 2012) and fuzzy logic (Ruitenbeek et al., 1999) or Bayesian belief networks (Barton et al., 2008).

A critical issue in the science-policy arena for diffuse agricultural water pollution and its cost-effective control is that there is growing evidence that the existing delivery of mitigation measures is not sufficiently targeted to deliver environmental outcomes commensurate with the value of environmental assets to society (Poole et al., 2013, Roebeling et al., 2016). In England, for example, a study of diffuse pollution and environmental status compliance concluded that the substantial expenditure on controlling the problem had not delivered value for money (NAO, 2010). Independent scientific evidence has also underscored the limited impact resulting from the current farmer uptake of water pollution interventions at national scale (Collins and Zhang, 2016). Consequently, packages of control measures need to be reviewed and revised to help secure positive environmental outcomes. Such experience is common across EU Member States and in its review of River Basin Management Plans (RBMPs) in 2012, the European Commission recommended that there is a need to ‘step up ambition in taking measures to achieve good status’.

Article 11.3 of the EU WFD sets out the requirements for PoMs to implement options and methods for preventing further deterioration of the status of freshwater environments. Control measures are divided into ‘basic’ (mandatory) and ‘supplementary’ (incentivised) categories. ‘Basic’ measures are described as minimum requirements including relevant existing EU legislation (e.g. the Nitrate Directive), designed to control practices resulting in point (e.g. farm yards) and diffuse (e.g. fields) source pollutant emissions. Mandatory expectations of farmers in England are outlined in so-called Cross Compliance which must be followed to secure support payments such as those administered by the Basic Payment Scheme (BPS) or agri-environment agreements. Cross Compliance (Defra, 2016) comprises Statutory Management Requirements (SMRs) and standards of Good Agricultural and Environmental Condition (GAEC). In terms of SMRs relevant to agricultural pollution control, SMR1 is most relevant and pertains to reducing water pollution in Nitrate Vulnerable Zones (NVZs designated under the Nitrate Directive). GAEC rule 1 (establishment of buffer strips along watercourses), GAEC 4 (minimum soil cover), GAEC 5 (minimum land management reflecting site specific conditions to limit soil erosion) and GAEC 6 (maintenance of soil organic matter level through appropriate practices, including a ban on burning arable stubble, except for plant health reasons) are all relevant to nutrient and sediment management by the agricultural sector in England.

To comply with Article 11.3 and in the context of the need to review and revise PoMs, the Department for Environment, Food and Rural Affairs (Defra) and Environment Agency in the UK have recently funded research to inform policy on the options to develop a new candidate set of ‘basic’ measures that address the most common causes of agricultural water pollution. These measures need to be broadly applicable to all farmers for helping to tackle harmful emissions, including those represented by nutrients and sediment. The uptake of these ‘basic’ measures could be encouraged through a range of approaches including, government sponsored advice, promotion by the industry, as well as inclusion in farm assurance schemes and Cross Compliance, with strategic implementation underpinned by a ‘polluter pays’ approach driven by regulation. The work aimed to identify a candidate revised set of ‘basic’ measures that would be effective in addressing the most common water quality pressures and, critically, to gauge its acceptability to the farming industry. In doing so, five steps were used in this study: i) examination of the main pollution pressures arising from agriculture; ii) assessment of the current regulatory expectations of farmers; iii) identification of an alternative set of ‘basic’ measures; iv) assessment of the technical costs and effectiveness of implementing the alternative set of ‘basic’ measures, and; v) consideration of the estimates of effectiveness in the context of cross sector pollutant emissions to rivers. The work involved integrating industry engagement and computer modelling of the technically feasible impacts of increased uptake of the shortlisted measures and therefore differed from much previous work wherein scientists independently select mitigation scenarios and run the corresponding simulations.

## 2 Methods

### 2.1 The overall rationale

To achieve the overarching objective of assessing the cost-effectiveness of a candidate set of ‘basic’ (mandatory) control measures for agricultural nutrient and sediment pollution across England, six specific research sub-objectives were established, viz:

1. To examine the available evidence on the contribution of agriculture to not meeting water quality standards across England to help inform selection of the candidate ‘basic’ measures that will contribute towards the achievement of environmental objectives.
2. To compare to what extent these evidence-based requirements are aligned with the current regulatory expectations of farmers (including those under existing environmental law and Cross Compliance requirements) as a preliminary screening of on-farm measures.
3. To identify an alternative set of ‘basic’ measures (specific control actions to be taken at farm level) unconstrained by current delivery mechanisms, based on scientific assessment and stakeholder consultation.
4. To optimise the selection of a candidate revised set of ‘basic’ measures using data from sentinel research catchments.
5. To assess the technical costs and effectiveness of implementing the candidate set of ‘basic’ measures at national scale across England.
6. To place the estimates of effectiveness in the context of cross sector pollutant emissions to rivers in order that the impacts of targeting the agricultural sector alone are better projected.

### 2.2 Assessment of the contribution of agriculture to not meeting WFD targets for water quality

An assessment of the contribution of agriculture to failure to meet WFD water quality targets was undertaken using the WFD Reasons for Failure (RFF) database (Environment Agency Reasons for Failure database v.27.06.2012).

### 2.3 Preliminary screening of potential on-farm ‘basic’ measures for nutrient and sediment control using WFD selection criteria

A list of mitigation measures that have the potential to address diffuse water pollution from agriculture was compiled from various key sources (Defra, 2010a, Defra, 2012, Newell Price et al., 2011, Schoumans et al., 2011, EU COST 869, n.d). The final list amounted to 708 individual mitigation measures which were assessed in a two-stage filtering process to first evaluate whether options could qualify as ‘basic’ measures against an agreed list of selection criteria, and secondly, whether the options would receive support from the agricultural industry in terms of their applicability and practicability of implementation. Selection criteria for the preliminary screening of the measures were agreed with the WFD Joint Implementation Group (Defra, Environment Agency, Natural England) against which the 708 measures could be assessed. On this basis, it was agreed that ‘basic’ control measures should be recognised as reflecting good farming practice, be effective at reducing losses of specified pollutants associated with the most common causes of water quality failing WFD standards (even if there was some risk of pollution swapping), be supported by evidence on performance, include methods that control diffuse losses but also small on-farm point sources, such as storage sites, and comply with the legal definitions in WFD Article 11.3.

### 2.4 Industry stakeholder scoring of ‘first filter’ on-farm ‘basic’ measures for nutrient and sediment control

Analysis of costs and effectiveness is a standard tool to help inform the development of PoMs (Trepel, 2010, Lam et al., 2011, Vinten et al., 2012), and such analysis is commonly combined with participatory approaches to gain insights and feedback from key stakeholders (Wright and Fritsch, 2011, Wright and Jacobsen, 2011, Perni and Martinez-Paz, 2013, Wilkinson et al., 2014, Collins et al., 2016). At an industry stakeholder workshop in London in March 2013, scoring of the ‘first filter’ measures for acceptability, practicability and applicability was undertaken. The workshop participants included representatives from the Agricultural Industries Federation (AIC), Allerton Trust, Association of Rivers Trusts, Agricultural and Horticultural Development Board (AHDB), Country Land and Business Association (CLA), Forestry Commission (FC), Farmers' Union of Wales (FUW), Game and Wildlife Conservation Trust (GWCT), National Farmers' Union of England and Wales (NFU), National Trust (NT), Pond Conservation Trust (now the Freshwater Habitats Trust), Royal Society for the Protection of Birds (RSPB), Tenant Farmers Association (TFA), Water UK (representing the water companies) and the Wildlife Trusts.

Through managed discussion, control measures were scored for i) engagement, commitment and ambition to support implementation, and; ii) the overall acceptability of the measures in terms of cost and practicability. Separate breakout groups scored each of the measures grouped in the following key categories: farmyard, surface and drainage infrastructure and management; field/soil/land management; nutrient/manure management planning and application; riparian management. The ‘first filter’ list of measures was assessed in terms of:

○ Uptake – what was the typical current level of uptake on farm (Low 0–30%; Moderate 30–60%; High 60–90%)?
○ Acceptability – How acceptable is the measure to the agricultural industry, levy bodies and unions? (1 – unacceptable to most farmers and to the industry; 2 – low acceptability – significant limitations to uptake due to low acceptability to farmers and the industry; 3 – acceptable to farmers keen to adopt this measure but without full support from the industry; 4 – the majority of farmers should do this with support from the industry and in some cases incentives; 5 – all farmers should do this – full support from the industry and no incentive/capital grant required).
○ Practicability – How likely is it that the measure would be implemented given other significant constraints on agricultural systems, including cost to the farm business or practical implementation resulting in significant loss of income or affecting business viability? (1 – totally impractical for most farmers; 2 – practical limitations for many farmers; 3 – practical difficulties could be overcome with incentives; 4 – a few practical limitations for some farmers – easily overcome; 5 – no practical limitations – easy to implement).
○ Applicability – How applicable is the measure to the range of farming systems across England in terms of factors such as farm type, agro-climatic region, soil type, livestock housing and manure storage? (1 – applicable to very few farms; 2 – not applicable to the majority of farming sectors/soil types; 3 – applicable to ~ 50% of the farming community/agricultural land; 4 – applicable to > 50% of the farming community/soil types; 5 – generally applicable to all farmers).

A ‘delivery potential index’ score was calculated to select a shorter list of ‘basic’ measures for which successful implementation would be more likely in the future. This ‘delivery potential index’ was calculated using the following procedure:

• Each measure was scored (1–5) in terms of acceptability to, practicability for, and applicability, across the agricultural industry.
• Any measure that scored ‘3’ or lower on acceptability (i.e. “Acceptable for farmers keen to adopt this measure but without positive support from industry”) was removed unless there were good grounds for including it (e.g. it is already part of Silage, Slurry and Agricultural Fuel Oil regulations; SSAFO) i.e. no legal requirements were removed due to lack of industry support.
• ‘Delivery potential index’ scores were summed for each measure based on the scores for acceptability, practicability and applicability.
• Any measure that (to the nearest whole number) scored < 7 out of 15 (i.e. < 5/10 on the adjusted score) was removed from further consideration.

### 2.5 Optimising the selection of a candidate set of on-farm ‘basic’ mitigation measures for nutrient and sediment control using the Demonstration Test Catchments (DTCs)

A modelling approach was used to shortlist the industry supported ‘first filter’ measures that, in combination, would technically be most effective in addressing nutrient and sediment pollution from agriculture and which therefore would be most usefully implemented as ‘basic’ control measures (i.e. be included in a final candidate set of ‘basic’ measures). The approach was founded on the use of the Excel-based decision support tool FARMSCOPER (FARM SCale Optimisation of Pollutant Emission Reductions) developed recently to help inform the management of diffuse agricultural pollution across England and Wales (Zhang et al., 2012, Gooday et al., 2014, Collins and Zhang, 2016, Collins et al., 2016, Zhang et al., 2017a, Zhang et al., 2017b). The work reported here differed from previous published studies using FARMSCOPER by integrating agri-industry and wider stakeholder (rather than farmers alone) scoring of measures and computer simulation of the technically feasible impacts of the measures shortlisted on that basis. The simulations used a new upscaling version of FARMSCOPER (Gooday et al., 2015), whereas previous published work was based on a preliminary framework incorporating replicate model farms but without automatic upscaling to landscape units. The computer simulations used FARMSCOPER since this is currently the leading policy tool for exploring diffuse pollution management scenarios in England and the science team was contracted to use it. FARMSCOPER is founded on a suite of well-established models which have all been used in national scale predictions for policy support. These models simulate nitrate, sediment, phosphorus, ammonia, methane and nitrous oxide emissions to the aquatic and atmospheric environments. Nitrate predictions are based on the NEAP-N model (Anthony et al., 1996). In the case of phosphorus and sediment, FARMSCOPER predictions use the Phosphorus and Sediment Yield CHaracterisation In Catchments (PSYCHIC) process-based model (Collins et al., 2007, Davison et al., 2008, Stromqvist et al., 2008, Collins et al., 2009, Comber et al., 2013, Collins and Zhang, 2016). Three principal soil types are represented in FARMSCOPER. These soil types were chosen to reflect the likelihood of agricultural under-drainage: permeable free draining soils; impermeable soils where artificial drainage is required to make them suitable for arable cultivation, and; impermeable soils where artificial drainage is required to make them suitable for either arable or grassland agriculture. These generic soil types provided a basis for simplifying the generation of pollutant export coefficients for farming systems on contrasting soils. Table S1 in the on-line SI shows the match between HOST (Hydrology of Soil Types; Boorman et al., 1995) classes and FARMSCOPER soil categories.

Agricultural management practice is simulated in FARMSCOPER using representative farm types (see Farm types section and Table S2 in on-line SI) derived from the Defra Robust Farm Type (RFT) classification scheme (Defra, 2010b), which is widely adopted in existing farm surveys undertaken across England and Wales. FARMSCOPER comprises a library of > 100 mitigation methods, each of which is characterised in terms of its impacts on pollutant emissions and the costs or savings that implementation of the method would incur for farmers. Impacts of multiple mitigation methods are multiplicative, such that the effectiveness of multiple methods targeting the same aspects of pollutant loss will be less than the sum of their individual impacts. Simulations generate outputs which include pollution swapping (reduction in the loss of one pollutant is associated with an increase in another) resulting from on-farm mitigation measures and avoidance of pollution swapping between emissions to water and air was a prerequisite for measure selection in this work. The costs (reference year 2013) of method implementation account for changes to the variable costs and gross margin of a crop or stock enterprise, changes to the fixed costs or overheads associated with labour and machinery and capital investment using a number of sources (e.g. Nix, 2009). Capital costs are typically amortised over 5 to 20 years, depending on the expected lifetime of the corresponding investment and any associated loans. Additional information on cost calculations is provided in the on-line SI.

Current or so-called business-as-usual (BAU) implementation of control measures is incorporated into FARMSCOPER to ensure that the technical potential for change in pollutant pressure in conjunction with any new theoretical package of measures is not over-estimated. Prior uptake represents various factors including the physiographic environment, farm type (i.e. applicability of a mitigation method) and the history of incentives via financial support or regulation. Estimates of prior implementation are expressed as a percentage of the applicable area or relevant livestock excreta on farm holdings. The assessment of prior implementation is described in detail in Gooday et al. (2015) – implementation rates are summarised on an indicator scale to provide an uncertainty range for the rates.

The estimates of average efficacy are lower than the central values of the ranges to provide a conservative assessment of measure impact. An additional distinction is made between measure uptake within and outside of NVZs since these have a regulatory Action Programme and although this is designed to target nitrate pollution, recent Defra Farm Practice Survey returns have collected some data which distinguish the uptake between NVZ and non-NVZ areas of some additional measures (e.g. management of grassland compaction) which can impact on nutrient and sediment loss. The efficacy of individual control measures in the FARMSCOPER library is based on literature reviews and elicitation of expert judgement (e.g. Newell Price et al., 2011, Cuttle et al., 2016). To help account for gaps in the empirical evidence base for some control measures and the range in efficacy values reported for the same measures by different studies, efficacy is summarised in FARMSCOPER on an indicator scale (Table S3).

Following consultation with the project Steering Group (Defra, Environment Agency, Natural England), it was decided that the Demonstration Test Catchments (DTCs; McGonigle et al., 2012, McGonigle et al., 2014), i.e. Hampshire Avon, Tamar, Eden and Wensum (Fig. S1), would be used as representative case study areas for the initial modelling runs. These sentinel research landscapes capture 87% of the rainfall/soil combinations across England plus all major farm types. For the model simulations, only those rainfall/soil combinations representing > 5% of the corresponding frequency distribution for each DTC were included. Table S4 summarises the soil group, rainfall band and RFT combinations for each DTC. A sub-set of the RFTs represented in FARMSCOPER was chosen, considering the robustness of available data and their significance for agricultural diffuse pollution nationally across England. On this basis, horticulture and poultry farms were excluded from further analysis in the model runs based on the DTCs.

Detailed crop and grass areas as well as livestock numbers from the 2010 June Agricultural Survey (JAS) were used to populate the data required by FARMSCOPER for the representative farm types in each DTC (Table S4). Farm type specific fertiliser application rates were based on the 2010 British Survey of Fertiliser Practice (BSFP Authority, 2011, BSFP Authority, 2013). Default manure management practices for each farm type in FARMSCOPER were used without modification. Various data sources were used to establish the BAU implementation of the ‘first filter’ measures in the DTCs. These included a ‘snap-shot’ of active agri-environment schemes in March 2012 at WFD Water Management Catchment (WMC) scale provided by Natural England, baseline farm survey data collected by the DTC programme, the proportion of each DTC that was in a designated NVZ (Tamar < 1% of utilised agricultural area (UAA), Eden 17%, Hampshire Avon 85% and Wensum 81% UAA) and expert judgement based on farm visits and national surveys including the BSFP and Farm Practices Surveys (2009–11).

FARMSCOPER can be used to optimise the selection of on-farm control measures to identify a best set using a prerequisite criterion. Previous work has used genetic algorithms for such optimisation (e.g. Veith et al., 2003) and FARMSCOPER uses the NSGA-II algorithm (Deb et al., 2002) for this purpose given its wide uptake and use in comparison of computational search techniques (Coello et al., 2007). The objective function, which was based on a pre-determined level of water pollutant loss reduction with no concomitant increase in gaseous emissions, was set for individual farms rather than per catchment or region and no interactions between farms were modelled. The objective function did not include a stipulation relating to control measure cost. Optimisation runs were used to assess the combined impact of the parameterised ‘first filter’ measures on BAU nutrient and sediment loadings delivered to rivers from agriculture. Measures were then ranked based on the number of times each individual option was included in FARMSCOPER-determined optimum measure combinations to deliver a set of water pollutant reduction targets agreed with the project Steering Group, in this case, a minimum 2% reduction in nitrate, phosphorus and sediment losses with no unintended increase in losses of other pollutants (i.e. gaseous) represented in the FARMSCOPER tool (i.e. minimal ‘pollution swapping’). On this basis, it was therefore the number of times that a measure is selected for inclusion in optimal measure combinations that determined its ranking rather than any particular cost-effectiveness ranking, which is likely to reflect each combination of agro-climate, soil type and RFT, as well as the level of BAU implementation. Optimisation runs used a population size of 50 and a generation value of 100; these are based on tests for settings capable of generating stable solutions during the development of the model. Scientifically, the low threshold (minimum 2%) for water pollutant reductions related to emission reductions in addition to those currently achieved under business-as-usual uptake of on-farm control measures which is typically ~ 10–15% or less. The stipulation of revised ‘basic’ measures is more about delivering widespread general improvements to business-as-usual reductions in diffuse pollution from agriculture in conjunction with EU Pillar I funding, rather than delivering high reduction rates but more geographically restricted to specific farm types. For the FARMSCOPER optimisation runs, there were 28 farm-soil-climate type combinations in the Eden DTC; 24 in the Wensum; 16 in the Avon; and 30 in the Tamar DTC. Control measures included in the selected measure combinations were pooled by DTC and their frequencies counted.

### 2.6 Simulating the costs and efficacy of the candidate set of on-farm ‘basic’ control measures for nutrient and sediment abatement at the Water Management Catchment (WMC) scale

The most recent version of FARMSCOPER (Gooday et al., 2015) incorporates an additional tool that automates the generation and assessment of pollutant losses and measures impacts for multiple farms to represent one or more catchments, using data that is appropriate for inclusion in a publicly available tool. The approach requires the number of farms of each RFT found in a catchment on any of the six rainfall zones and three soil types recognised by FARMSCOPER. The total cropping and livestock within the catchment is then distributed across these farms based upon the relative likelihood of occurrence of the different crops and livestock on the different farm types, derived from national data, and assumptions on typical stocking rates. FARMSCOPER contains the data from the 2010 JAS required to simulate the 92 Water Management Catchments found in England (Fig. S2). The required nitrogen and phosphorus fertiliser data were taken from the 2010 British Survey of Fertiliser Practice (BSFP Authority, 2011), which provides crop-specific rates by farm type. FARMSCOPER was used to estimate the BAU emissions and the implementation costs and efficacy of nutrient and sediment reduction by the ‘basic’ measures at WMC scale, accounting for any current implementation of these measures reducing the potential for future implementation. Scenario analysis assumed a flat rate 95% future implementation of the ‘basic’ measures by the appropriate farming systems as this is the upper ceiling used by the policy teams to which this work was delivered. On this basis, relevant measures were applied to relevant farms rather than shortlisted measures being applied generically to all farms regardless of their structure (e.g. presence of livestock). To assess the impacts of the uncertainty in the estimates of current implementation, the calculations of ‘basic’ measure costs and efficacy were made using the average current implementation rate and the upper and lower bounds.

This scaling up approach has been found to produce pollutant losses at WMC scale that are consistent with the original predictions of the source input models (Gooday et al., 2015). National scale FARMSCOPER simulations of BAU emissions to water from agriculture have previously been evaluated using PARCOM (1991–2010) monitoring (Collins et al., 2016, Zhang et al., 2017a) and Harmonised Monitoring Scheme (HMS) data (1980–2010) collected at 33 sites (Zhang et al., 2017b). These evaluation exercises demonstrate that the tool is able to simulate regional variations in pollutant pressures, but with the fits between modelled and monitored data being better for nitrate than sediment and phosphorus.

### 2.7 Correcting the WMC scale predictions for the impact of the candidate set of on-farm ‘basic’ mitigation measures for nutrient and sediment control using cross sector source apportionment

In projecting the technically feasible impact of theoretical intervention scenarios targeting agriculture only, it is important to factor in cross sector source apportionment information to provide a better reflection of impact on in-stream pollutant loads. Here, the SEPARATE (SEctor Pollutant AppoRtionment for the AquaTic Environment) screening tool (Zhang et al., 2014) was used to generate estimates of nutrient and sediment source apportionment for each WMC. The pollutant sources included in SEPARATE comprise agriculture, urban areas, channel banks, direct atmospheric deposition, sewage treatment works, septic tanks, combined storm overflows, storm tanks and groundwater. Different screening tools are reported in the literature for both the UK (e.g. Comber et al., 2013) and elsewhere (e.g. Giupponi and Vladimirova, 2006, Brouwer and De Blois, 2008, OECD, 2012). SEPARATE was selected since it includes river bank erosion which can be an important source of sediment which was one of the water pollutants considered in this work.

## 3 Results and discussion

### 3.1 Assessment of the contribution of agriculture to not meeting WFD targets for water quality

Diffuse water pollution from agriculture is a significant reason for failure of water bodies across England and Wales in meeting WFD ‘good ecological status’. By way of example, of all the WFD Reasons for Failure (RFF) in England and Wales recorded by the Environment Agency in 2012, 18% were attributed to agriculture (Environment Agency Reasons for Failure database v.27.06.2012). The main agricultural pressures resulting in water quality failures in 2012 were associated with diffuse water pollution from agriculture (DWPA; 88% of the agricultural contribution; Environment Agency Reasons for Failure database v.27.06.2012). In terms of activities resulting in significant diffuse water pollution from agriculture, the most important identifiable on-farm sources were arable fields (26%), dairy/beef fields (13%) and mixed agricultural runoff (21–24%). However, the specific agricultural activity that resulted in water quality failure was not identified in 32% of cases (Environment Agency Reasons for Failure database v.27.06.2012). Nevertheless, the survey data in the Reasons for Failure database suggested that improved management of arable and grass fields and farmyards could deliver positive impact in helping to reduce water quality failures currently attributable to agricultural runoff. Table 1 summarises the relative contributions of specific pollutants to the agricultural pressures on water bodies across England and Wales. These estimates, again based on the Reasons for Failure database, suggested that the primary agricultural pollutants requiring improved mitigation were sediment, phosphate and nitrate. ‘Basic’ control measures therefore need to be particularly relevant to controlling these emissions.

**Table 1:** The relative contributions of specific pollutants to agricultural Reasons for Failure (Environment Agency Reasons for Failure database v.27.06.2012).

Pollutant	Relative contribution to agricultural reason for failure (%)
Sediment	67
Phosphate	37
Nitrate	33
Dissolved oxygen	24
Ammonia	15

### 3.2 Preliminary screening of potential (n = 708 to n = 90) on-farm ‘basic’ measures for nutrient and sediment control using WFD selection criteria

The assessment of the 708 potential measures by the project scientific team against agreed selection criteria resulted in a ‘first filter’ short list of 90 control measures (Table S5) for nutrient and sediment control, which were sorted into the following key categories (the number of measures in each category is provided in brackets): farmyard, surface and drainage infrastructure and management (23); field/soil/land management (40); nutrient/manure management planning and application (20), and; riparian management (7). Measures that were existing 2009 Nitrate Vulnerable Zone (NVZ) Action Programme rules could, as ‘basic’ measures, potentially become mandatory outside NVZs or be introduced on an initially voluntary basis. The outcomes of the ‘first filter’ (n = 90) were signed off by policy team members on the project steering committee.

### 3.3 Industry stakeholder scoring of ‘first filter’ (n = 90 to n = 63) on-farm ‘basic’ measures for nutrient and sediment control

Scoring of the ‘first filter’ (n = 90) measures for acceptability, practicability and applicability reduced the number of control measures to 63. Those measures identified as being unacceptable are highlighted in Table S5. It should be noted, however, that at the workshop, the principal industry stakeholders were represented by a small number (< 30) of individuals. The views of other members within the represented organisations and of individual farmers will inevitably vary (sometimes significantly) from those expressed at the workshop.

### 3.4 Optimisation results for identifying the candidate set (n = 12) of ‘basic measures’ for nutrient and sediment abatement using the Demonstration Test Catchments (DTCs)

The measures listed in Table S5 that are parameterised within the FARMSCOPER decision support tool were identified (Table 2) and included in modelling runs for costs and efficacy. FARMSCOPER runs were therefore not able to represent all ‘basic’ measures (particularly those that control small point sources) identified by the ‘first filter’ based on stakeholder consultation, but did provide a good indication of the overall typical costs and effectiveness of combinations of measures. Although some measures could not be modelled using FARMSCOPER, these act on the same pollutants and in the same pathway as measures present in the FARMSCOPER modelling framework and thus lend support to using this tool. For example, ‘rotate stock more frequently to reduce risk of poaching’ is not parameterised within FARMSCOPER, but has some relationship to ‘reduce field stocking rates when soils are wet’ or ‘locate out-wintered stock away from watercourses’, which are both included.

**Table 2:** Industry supported ‘first filter’ measures included in the FARMSCOPER modelling framework (those options with * were covered under 2012 regulation or Statutory Management Rules (SMRs, e.g. NVZ rules)).

Control measures	Typical efficacy for pollutant reductions (ranges included where possible)		
Farmyard surface and drainage infrastructure and management	Nitrate	Phosphorus	Sediment
Farm track management	2	2	2
Field/soil/land management			
Irrigate crops to achieve maximum yield	10		
Reduce field stocking rates when soils are wet	− 2–10	− 2–10	10
Move feeders at regular intervals	10	10	10
Leave over winter stubbles	10	25	10
Manage over-winter tramlines	25	25	25
Establish cover crops in the autumn	50	80	80
Reduce the length of the grazing day/grazing season	− 10–25	− 10–25	25
Avoid irrigating at high risk times	2	2	2
Nutrient/manure management planning and application			
Fertiliser spreader calibration	2	-	-
Do not apply manufactured fertiliser to high-risk areas⁎	10–25	25	-
Avoid spreading manufactured fertiliser to fields at high-risk times⁎	2	10	-
Do not spread slurry or poultry manure at high-risk times⁎	25	25	-
Increase the capacity of farm slurry stores to improve timing of slurry applications⁎	10	10	-
Do not apply P fertilisers to high P index soils	-	25	-
Do not apply manure to high-risk areas⁎	25	25	-
Incorporate manure into the soil⁎	− 10–25	50	-
Use a fertiliser recommendation system	10	2	-
Riparian management			
Locate out-wintered stock away from watercourses	2	2	2
Establish and maintain artificial wetlands - steading runoff	25	50	-
Site solid manure heaps away from watercourses/field drains⁎	10	10	-
Intensive ditch management on arable land	− 2	− 2	− 2
Intensive ditch management on grassland	− 2	− 2	− 2
Establish riparian buffer strips	2–10	2–50	2–50

- Control measures do not impact on the pollutant in question.

⁎ Measures covered under 2012 regulation or statutory management requirements (SMRs e.g. NVZ Action Programme rules).

Summary statistics of the results for the technical efficacy and costs of the modelled ‘first filter’ measures applied to the representative farms in each DTC are presented in Table 3. Non-parametric statistics (e.g. medians and quartile ranges) were calculated as most of the sample populations had non-normal frequency distributions. In the Avon DTC, the total annual costs of implementing the ‘first filter’ control measures were predicted to range between £3328 and £10,467 per farm, compared to corresponding ranges of £693–£3539 in the Eden, £848–£7696 in the Tamar and £4698–£12,873 in the Wensum DTC. The predicted uncertainty ranges in the reductions of nitrate pollution per farm varied from 2.2–3.3% (Wensum) to 3.4–5.9% (Tamar). The corresponding ranges for phosphorus per farm varied from 5.7–7.6% (Wensum) to 4.9–9.1% (Eden) and for sediment from 1.2–3.7% (Wensum) to 2.2–10.3% (Tamar). Across the DTCs, total predicted annual costs for implementing the ‘first filter’ measures ranged from £834 to £7624 per farm, with corresponding pollutant reduction uncertainty ranges of 2.8–5.4% (nitrate), 5.6–9.4% (phosphorus) and 1.3–7.4% (sediment).

**Table 3:** The technically feasible impact of the implementation of modelled ‘first filter’ measures for nutrient and sediment control in the Demonstration Test Catchments (DTCs)^a^, ^b^.

DTC	Statistic	Total cost	Nitrate	Phosphorus	Sediment
		£	%	%	%
Avon	Q1	3328	2.7	6.3	1.7
	Q3	10,467	3.8	8.7	7.3
	Median	6622	3.3	7.2	2.7
Eden	Q1	693	3.8	4.9	1.0
	Q3	3539	5.7	9.1	7.8
	Median	1460	4.5	7.5	5.3
Tamar	Q1	848	3.4	6.2	2.2
	Q3	7696	5.9	11.2	10.3
	Median	3148	4.5	9.6	5.2
Wensum	Q1	4698	2.2	5.7	1.2
	Q3	12,873	3.3	7.6	3.7
	Median	6129	2.7	6.8	2.6
All DTCs	Q1	834	2.8	5.6	1.3
	Q3	7624	5.4	9.4	7.4
	Median	4181	3.8	7.4	3.6

a All pollutant values in the table represent percentage decreases in annual losses at the farm scale relative to loadings associated with BAU.

b The median and quartile data are calculated for the same groups of cases and the costs to farmers and pollutant reductions equally correspond to the same groups of predictions.

The relative frequency of selection of the modelled ‘first filter’ measures in the optimised combinations for the DTCs is presented in Table 4. There was little variation between the DTCs. ‘Farm track management’, ‘irrigate crops to achieve maximum yield’ and ‘intensive ditch management on grassland’ were not selected in any of the optimisation runs due to higher implementation costs (and therefore lower cost-effectiveness) or because other measures could deliver similar or greater reductions in BAU pollutant loadings at reduced cost. Those control measures with the highest counts (Table 4) in the optimisation runs are applicable to a higher proportion of farms in the DTCs than those options with low counts, and are most likely, on average, to reduce multiple pollutants simultaneously with lower annual costs to the farms. The options listed in Table 5 were selected as the candidate set (n = 12) of ‘basic’ measures for controlling nutrient and sediment emissions from agricultural land across England. All 12 measures had high ‘delivery potential’, meaning that some measures which were selected frequently (Table 4) in the optimisation runs (e.g. ‘establish cover crops in the autumn’) were not included in the final shortlist (Table 5). ‘Use a fertiliser recommendation system’, ‘do not apply manufactured P fertilisers to high P index soils (with an Olsen P index of 4 or above)’, ‘move feeders at regular intervals’ and ‘leave over-winter stubbles’ are not covered by the current regulatory baseline, but the first of these measures is an Action Programme rule for NVZs. A further 7 of the measures in the candidate set (n = 12) of ‘basic’ control measures are also NVZ rules and therefore uptake outside of NVZ designations would be in addition to the current regulatory landscape (Table 5).

**Table 4:** The percentage (%) of optimisation runs for which each modelled ‘first filter’ measure was included in optimal measure combinations to meet the prescribed pollutant load reduction targets of 2% in the Demonstration Test Catchments (DTCs).

Control measure	Avon	Eden	Tamar	Wensum	All DTCs
Use a fertiliser recommendation system	19	16	17	17	17
Do not apply manufactured P fertilisers to high P index soils (with an Olsen soil P index of 4 or above)	17	15	15	14	15
Establish cover crops in the autumn	6	11	11	17	13
Site solid manure heaps away from watercourses/field drains^a^	4	3	3	11	6
Do not apply manufactured fertiliser to high-risk areas^a^	4	8	7	2	5
Do not spread slurry or poultry manure at high-risk times^a^	6	5	4	4	4
Do not apply manure to high-risk areas^a^	4	5	4	4	4
Establish riparian buffer strips	3	6	5	1	4
Fertiliser spreader calibration^a^	2	3	5	3	4
Manage over-winter tramlines	1	6	3	2	3
Reduce field stocking rates when soils are wet	1	0	4	7	3
Reduce the length of the grazing day/grazing season	4	3	2	7	3
Avoid spreading manufactured fertiliser to fields at high-risk times^a^	4	3	3	1	3
Increase the capacity of farm slurry stores to improve timing of slurry applications^a^	1	0	3	4	3
Locate out-wintered stock away from watercourses	0	4	3	1	3
Incorporate manure into the soil^a^	1	3	2	0	2
Move feeders at regular intervals	0	0	2	2	1
Fertiliser sprayer calibration	2	3	0	1	1
Establish and maintain artificial wetlands - steading runoff	1	0	1	1	1
Intensive ditch management on arable land	0	0	2	0	1
Avoid irrigating at high risk times	0	0	1	1	1
Intensive ditch management on grassland	0	0	0	0	0

a Measures covered under 2012 regulation or statutory management requirements (SMRs e.g. NVZ Action Programme rules).

**Table 5:** The highest ranked measures identified using the optimisation runs for the Demonstration Test Catchments (DTCs).

1. Use a fertiliser recommendation system*
2. Do not apply manufactured P fertilisers to high P index soils (with an Olsen soil P index of 4 or above)
3. Move feeders at regular intervals
4. Leave over winter-stubbles
5. Do not apply manufactured fertiliser to high-risk areas*
6. Site solid manure heaps away from watercourses/field drains*
7. No overgrazing of natural or semi-natural grassland (GAEC 9)
8. Do not spread slurry or poultry manure at high-risk times*
9. Do not apply manure to high-risk areas*
10. Increase the capacity of farm slurry stores to improve timing of slurry applications*
11. Incorporate manure into the soil*
12. Avoid spreading manufactured fertiliser to fields at high-risk times*

Underlined measures are not covered by current regulatory controls and so would be an addition to the current requirement of farmers in any list of ‘basic’ measures. The remaining measures are either included in 2009 NVZ Action Programme rules (and therefore mandatory adoption outside NVZs would also be additional to the current regulatory landscape - * denotes NVZ AP rules), or are part of the current Cross Compliance regime – Good Agricultural and Ecological Condition (GAEC) rules.

### 3.5 Predicted costs and efficacy of the candidate set (n = 12) of ‘basic’ measures at national and WMC scale

**Fig. 1:**
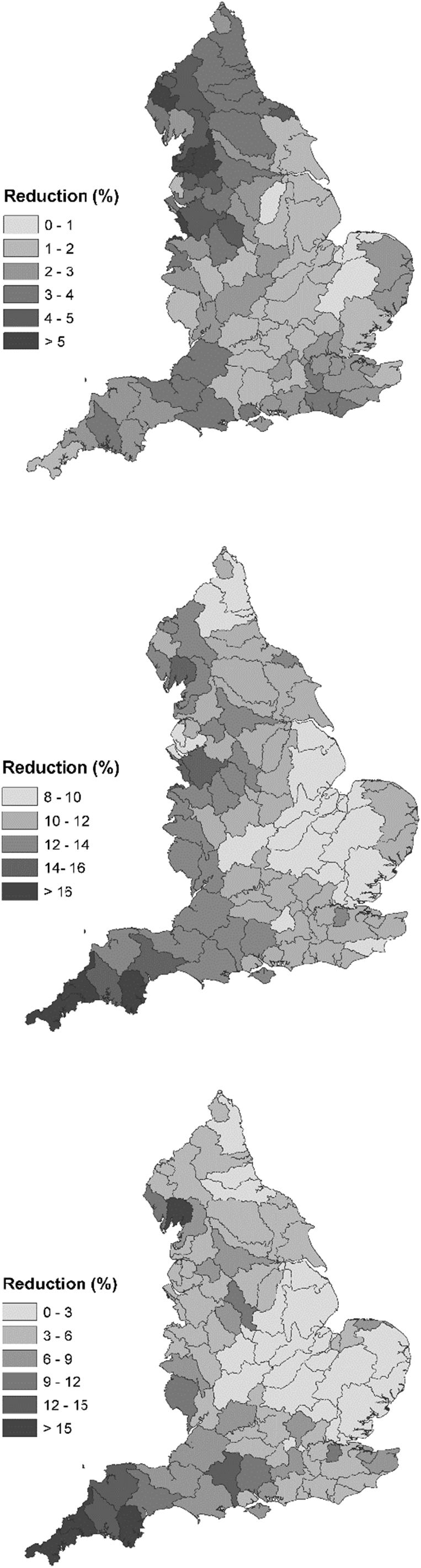
Projected impacts of the suite of candidate ‘basic’ measures on agricultural loads (upper - nitrate, middle - phosphorus and lower – sediment) for the WMCs across England.

The results of the WMC scale modelling, when summarised for the whole of England are shown in Table 6. Reductions in the national agricultural nitrate load are 2.5%, whilst corresponding reductions in phosphorus and sediment are 11.9% and 5.6%, respectively. These forecast reductions are less than those reported by previous papers using FARMSCOPER (e.g. Collins et al., 2016, Zhang et al., 2017a, Zhang et al., 2017b) but it is important to bear in mind those studies assessed larger suites of measures targeting, for example, farmer-preferred measures or interventions appropriate for the different components of the pollutant cascade. There is considerable spatial variation in the reductions at WMC scale (Fig. 1), with reductions in nitrate ranging from under 1% in Eastern England to over 5% in North-Western England. There is a similar spatial pattern in the reductions for phosphorus and sediment, with reductions lowest in the East (~ 10% for phosphorus and under 3% for sediment) and highest in the West (over 12% for phosphorus and over 6% for sediment). These reductions are comparable to those found for the DTCs, which show the same spatial pattern with lowest values in the Wensum and highest values in the Eden and Tamar. The relevance of the shortlisted (n = 12) control measures to farms with arable crops or livestock, or both, will be a factor here, since arable cropping dominates in the east and livestock farming in the west and more of the measures in the final shortlist are relevant to livestock systems. The total cost of applying these ‘basic’ measures across the whole of England was estimated to be £450 million per annum, which is equivalent to just over £50 per hectare of agricultural land. Readers are reminded that the selection of ‘win-win’ measures only was not stipulated for this work.

**Table 6:** Impact of the suite (n = 12) of ‘basic’ measures on national agricultural pollution loads assuming low, average and high current measure implementation^a^.

Current implementation	Nitrate	Phosphorus	Sediment	Total cost	Total cost
	%	%	%	(£m)	(£/ha)
Low	3.6	12.6	6.5	662	76
Average	2.5	11.9	5.6	451	52
High	1.9	10.8	4.1	265	30

a All pollutant values in the table represent percentage decreases in annual losses at the farm scale relative to loadings associated with BAU.

**Fig. 2:**
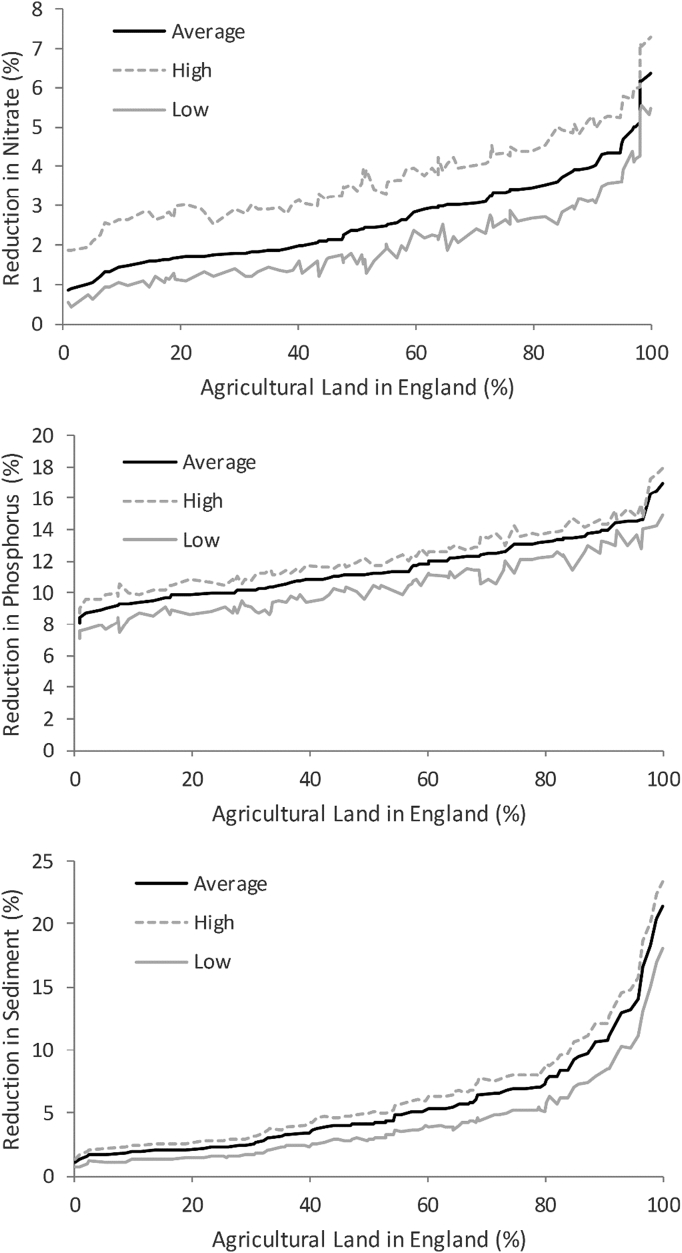
Effect of the uncertainty in estimates of BAU measure implementation on the potential for further reductions in nutrient and sediment emissions to water due to the candidate ‘basic’ measures.

Accounting for the uncertainty in the estimates of BAU measure uptake and, thus, the gap to be closed with full implementation of the ‘basic’ (n = 12) measures, shows that the potential national impacts of the ‘basic’ measures could be approximately 1% higher or lower than the average estimate (Table 6). The proportional uncertainty is greatest for nitrate (due to the low average estimate) and lowest for phosphorus (where the average estimate is highest). The national cost of ‘basic’ (n = 12) measure implementation changes by over 40% when uncertainty is incorporated. Fig. 2 presents the spatial variation in pollutant reductions estimated from this uncertainty analysis. Absolute changes in efficacy (related to uncertainty) can be large where the average estimate of impact was large (a change of up to 5.5% for sediment in areas where the average reduction was 20%), but proportional changes are highest when predicted average reductions are lowest (nitrate reductions ranging from 2% to 0.5% with an average of 1%). Given the spatial trend in reductions (i.e. greatest in the West and lowest in the East), the absolute uncertainty is thus highest in the East. Overall, the predicted impacts inclusive of uncertainty surrounding BAU measure implementation, exhibit limited variation, thereby suggesting that the inherent uncertainty in current measure uptake rates would not result in significantly variable impacts on agricultural load reductions.

### 3.6 Predicted efficacy of the candidate set (n = 12) of ‘basic’ measures at WMC scale corrected for cross sector source apportionment

**Fig. 3:**
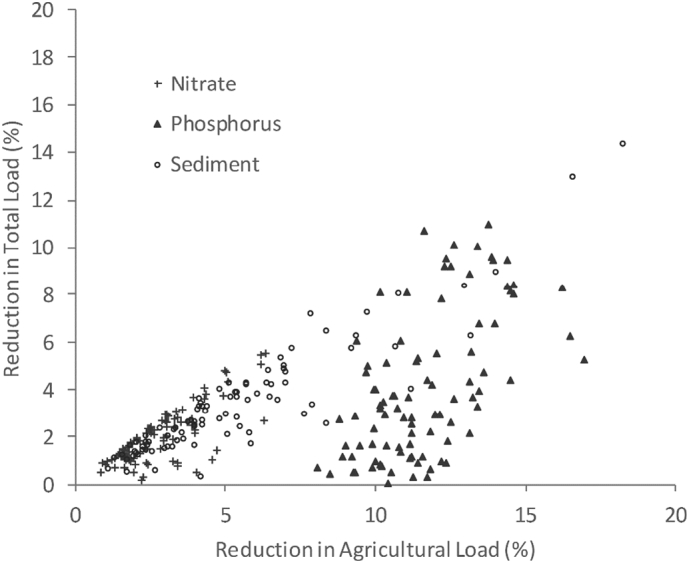
Reductions in the agricultural pollutant load due to the candidate ‘basic’ measures versus the reduction in the total pollutant load after accounting for the contributions from non-agricultural sectors.

The overall impacts of reductions in the agricultural pollutant load due to the candidate (n = 12) ‘basic’ measures will be reduced where agriculture is not the dominant source of pollution. Maps of the agricultural contribution to pollutants loads by WMC are contained in SI (Fig. S3). Fig. 3 shows that the predicted reductions in nitrate are generally only slightly lower when the sector apportionment is taken into account, which is because agriculture contributes 70% of the national nitrate load delivered to inland watercourses across England (Zhang et al., 2014). Fig. S4, as a supplement to Fig. 3, presents national maps of predicted reductions in the loads of each pollutant delivered to rivers, taking account of cross sector source apportionment. There are very large decreases in nitrate reductions in some WMCs where other sources dominate (typically due to major sewage treatment works near large urban areas). Agriculture also contributes approximately 70% of the national sediment load, but the non-agricultural contribution is not as localised as for nitrate and so most overall reductions are lower than predicted for agriculture alone (Fig. 3). Non-agricultural sources dominate the national phosphorus budget (72%) and so the overall effectiveness of the candidate (n = 12) ‘basic’ measures for phosphorus is much lower than that predicted for nitrate and sediment, although some WMCs still have reductions of around 10% (Fig. 3).

### 3.7 Policy implications

Agricultural diffuse pollution has been referred to as the ‘wicked problem’ given the inherent complexity for abatement arising from multiple pollutant sources, drivers, actors and environmental outcomes (Gunningham and Sinclair, 2005, Patterson et al., 2013). Top-down policy approaches to devising suites of on-farm measures can encounter various challenges and problems for political legitimacy ranging from shortcomings in tackling local environmental objectives using strategic generic solutions to legal issues surrounding on-ground implementation (Jacobsen et al., 2017). Current water quality policy for tackling agricultural diffuse pollution in England comprises a mixture of mandatory regulatory requirements (Cross Compliance), targeted regulation (e.g. NVZs), advice to support voluntary uptake of measures (e.g. CSF) and targeted incentivisation (the new Countryside Stewardship agri-environment scheme). The decision of the UK to depart the European Union means that its agricultural policy post 2020 might look substantially different. Ongoing debates are focussing on a number of policy options post the Common Agricultural Policy, including exit bonds as one off payments to help cushion farmers from the break from subsidies and an ecosystem services approach to incentivisation. Regardless of the overarching agricultural policy framework, however, it makes sense for some mandatory measures for diffuse pollution abatement to remain in the legal framework as a means of directing minimal performance requirements of farmers. The work reported here underpinned a Public Consultation in England during 2016 on revisions to existing Cross Compliance and there are plans for revised ‘basic’ measure requirements to be formally introduced in 2018.

### 3.8 Limitations of the work

Only a limited range of stakeholders attended the industry workshop to score the ‘first filter’ (n = 90 reduced to n = 63) list of measures, but given the time constraints associated with the policy development cycle, there was no opportunity to run repeat events. The farm business structure data used for the optimisation modelling in the DTCs was collected from the target experimental sub-catchments within these sentinel drainage basins meaning that the survey sample was typically 5% or less of the total population of farms within each DTC. The modelling approach used in this work inevitably has some limitations and uncertainties (see Collins et al., 2016, Collins and Zhang, 2016, Zhang et al., 2017a, Zhang et al., 2017b). FARMSCOPER simulates nitrate rather than total nitrogen loading, which underestimates the total diffuse nitrogenous pressure on waters in livestock farming regions (Durand et al., 2011). The specific diffuse N forms not included in the modelling exercise reported here therefore include the particulate and dissolved organic N delivered to waters from livestock wastes which generate both N enrichment and organic pollution impacts in streams, together with ammoniacal and nitrite, both of which are toxic at low concentrations to aquatic organisms (Durand et al., 2011). Nitrate was used in the work reported herein given that there is a drinking water target for nitrate, and in the absence of an ecologically-relevant threshold for total nitrogen in current UK water policy. FARMSCOPER maps measures using the presence of relevant cropping or livestock – for example, those control measures pertaining to livestock wastes are mapped onto farms with livestock, those pertaining to arable sources are mapped onto farms with arable crops. On the ground, the selection of measures should be location specific following detailed risk assessment, but here, a national modelling exercise has been used to simulate the potential impact of revised (n = 12) ‘basic’ control measure implementation using generic mitigation measure applicability to farm types. Although a flat future implementation rate of 95% was assumed for the ‘basic’ measures, as requested by the policy teams, in reality, there is likely to be variation among the upper uptake rates achievable for individual on-farm interventions included in any policy instrument due to a variety of reasons including the variable costs and any related barriers to uptake. Current uptake rates can limit the projected impact of future implementation where business-as-usual rates are already high (e.g. ‘use a fertiliser recommendation system’). Even where measures are stipulated by regulation, high compliance rates can be limited by numerous factors, despite the clear rationality of the measures in question, including farmers opting to decrease production intensity in the presence of a cross compliance scheme (e.g. Finger and Calanca, 2011). Uncertainty about the median outputs of the model simulations has been quantified using the inter-quartile range, given the nature of the distributions. It should be acknowledged, however, that this approach focusses on the central 50% of predictions rather than on all possible solutions. There are numerous aspects of uncertainty in predicting the overall cost-effectiveness of increased mitigation measure uptake that could be investigated, including the efficiency of measure implementation, the design aspects of implementation (with associated changes in costs and effectiveness) and the costs of implementation. Predicted effectiveness of measures can also be impacted by climate change, due to changes in baseline pollutant risks (which may increase or decrease, depending upon pollutant and location; Whitehead et al., 2009, Bussi et al., 2017, Zessner et al., 2017), changes in land use as a result of climate change or evolving policy drivers and changes in mitigation efficiency under different weather (e.g. precipitation) extremes. Investigation of all aspects of uncertainty for model simulations involving multiple mitigation measures was beyond the scope of this paper but, could be undertaken using one-factor-at-a-time (OFAT) analysis. In this context, the work reported here focussed on assessing the impact of one factor, uncertainty in baseline implementation, since consideration of a single aspect of uncertainty permitted estimation of its impact on model predictions. The uncertainty of the model simulations should be borne in mind when interpreting the outputs of the optimisation runs using the 2% criterion for pollutant reduction. Readers are reminded that the modelling framework simulates impact of mitigation measures on pollutant delivery to watercourses and does not therefore include consideration of wider knock-on effects such as reduced sediment delivery impacting on downstream ecosystems such as estuaries or lagoons. Although FARMSCOPER takes account of pollutant swapping, it does not simulate interactions (e.g. synergistic, antagonistic) between multiple stressors on end-points such as aquatic ecology. Even though the mitigation costs to farmers are armortised, the modelling work reported here focussed on reductions in nutrient and sediment delivery to watercourses from agriculture and did not include consideration of societal cost-benefit associated with the expected time lag between increased measure uptake on farms and the realisation of sustained improvements in water quality. Time lags are associated with numerous intermediate processes including those acting in long-term nutrient stores distributed across landscapes. It is clearly an unrealistic expectation of a national modelling exercise to represent high resolution location-specific mitigation measure applicability without detailed characterisation of field by field risks and indeed the attitudes/constraints of individual farmers at national scale. Nutrient and sediment delivery from farms to rivers is typically highly episodic in conjunction with storm events. The process-based models underpinning FARMSCOPER pollutant pressures are based on coarser resolution time-steps (monthly to annual), but the existing evidence base for the efficacy of on-farm measures for diffuse pollution control is commonly reported in terms of annual rather than storm scale impacts. Significant runoff events, especially in conjunction with shifts in rainfall regimes (e.g. Burt et al., 2016) clearly have the capacity to reduce the efficacy of on-farm measures over short time-steps, but the modelling work sought to characterise measure efficacy for typical current climatic conditions. The authors are unaware of any extensive information or, more particularly, any collation of such information on storm scale impacts of the entire suite of on-farm measures included in the work reported here.

## 4 Conclusion

The work herein demonstrates how a combination of review of available options, stakeholder discussion and ranking of individual measures and process-based modelling can be used to project the technically feasible impacts of alternative farming futures on nutrient and sediment emissions to watercourses across England. In the context of the decision of the UK to depart the European Union, there remains widespread recognition that mandatory ‘basic’ measures should be retained as part of the mix of policies designed to protect aquatic resources.

## Appendix A Supplementary data

Supplementary material Image 2

## References

[bb0005] Anthony S.G., Quinn P., Lord E. (1996). Catchment scale modelling of nitrate leaching. Asp. Appl. Biol..

[bb0010] Azzaino Z., Conrad J.M., Ferraro P.J. (2002). Optimizing the riparian buffer: Harold Brook in the Skaneateles Lake watershed, New York. Land Econ..

[bb0015] Bailey A., Deasy C., Quinton, Silgram M., Jackson B., Stevens C. (2013). Determining the cost of in-field mitigation options to reduce sediment and phosphorus loss. Land Use Policy.

[bb0020] Balana B.B., Vinten A., Slee B. (2011). A review on cost-effectiveness analysis of agri-environmental measures related to the EU WFD: key issues, methods and applications. Ecol. Econ..

[bb0025] Balana B.B., Jackson-Blake L., Martin-Ortega J., Dunn S. (2015). Integrated cost-effectiveness analysis of agri-environmental measures for water quality. J. Environ. Manag..

[bb0030] Barton D.N., Saloranta T., Moe S.J., Eggestad H.O., Huikka S. (2008). Bayesian belief networks as a meta-modelling tool in integrated river basin management — pros and cons in evaluating nutrient abatement decisions under uncertainty in Norwegian river basin. Ecol. Econ..

[bb0035] Baylis K., Peplow S., Rausser G., Simon L. (2008). Agri-environmental policies in the EU and United States: a comparison. Ecol. Econ..

[bb0040] Beddington, J.R., 2009. Food, energy, water and the climate: a perfect storm of global events? (Unpublished manuscript). www.bis.gov.uk/assets/goscience/docs/p/perfect-storm-paper.pdf.

[bb0045] Berbel J., Martin-Ortega J., Mesa P. (2011). A cost-effectiveness analysis of water-saving measures for the water framework directive: the case of the Guadalquivir River Basin in southern Spain. Water Resour. Manag..

[bb0050] Berger E., Haase P., Kuemmerlen M., Leps M., Schafer R.B., Sundermann A. (2017). Water quality variables and pollution sources shaping stream macroinvertebrate communities. Sci. Total Environ..

[bb0055] Boorman D., Hollis J., Lilly A. (1995). Hydrology of soil types: a hydrologically based classification of the soils of the United Kingdom. Institute of Hydrology Report No. 126, Wallingford, Oxfordshire.

[bb0060] Bouraoui F., Grizzetti B. (2014). Modelling mitigation options to reduce diffuse nitrogen water pollution from agriculture. Sci. Total Environ..

[bb0065] Brady M. (2003). The relative cost-efficiency of arable nitrogen management in Sweden. Ecol. Econ..

[bb0070] Brouwer R., De Blois C. (2008). Integrated modelling of risk and uncertainty underlying the cost and effectiveness of water quality measures. Environ. Model. Softw..

[bb0075] BSFP Authority (2011). The British Survey of Fertiliser Practice – Fertiliser Use on Farm Crops for Crop Year 2010.

[bb0080] BSFP Authority (2013). The British Survey of Fertiliser Practice – Fertiliser Use on Farm Crops for Crop Year 2012.

[bb0085] Burt T.P., Boardman J., Foster I., Howden N. (2016). More rain, less soil: long-term changes in rainfall intensity with climate change. Earth Surf. Process. Landf..

[bb0090] Bussi G., Janes V., Whitehead P.G., Dadson S.J., Holman I.P. (2017). Dynamic response of land use and river nutrient concentration to long-term climatic changes. Sci. Total Environ..

[bb0095] Cardenas L.M., Cuttle S.P., Crabtree B., Hopkins A., Shepherd A., Scholefield D., del Prado A. (2011). Cost effectiveness of nitrate leaching mitigation measures for grassland livestock systems at locations in England and Wales. Sci. Total Environ..

[bb0100] Chen Y., Shuai J., Zhang Z., Shi P., Tao F. (2014). Simulating the impact of watershed management for surface water quality protection: a case study on reducing inorganic nitrogen load at a watershed scale. Ecol. Eng..

[bb0105] Coello C., Lamont G., van Velduizen D. (2007). Evolutionary Algorithms for Solving Multi-objective Problems.

[bb0110] Collins A.L., Zhang Y. (2016). Exceedance of modern ‘background’ fine-grained sediment delivery to rivers due to current agricultural land use and uptake of water pollution mitigation options across England and Wales. Environ. Sci. Pol..

[bb0115] Collins A.L., Stromqvist J., Davison P.S., Lord E.I. (2007). Appraisal of phosphorus and sediment transfer in three pilot areas identified for the catchment sensitive farming initiative in England: application of the prototype PSYCHIC model. Soil Use Manag..

[bb0120] Collins A.L., Anthony S.G., Hawley J., Turner T. (2009). The potential impact of projected change in farming by 2015 on the importance of the agricultural sector as a sediment source in England and Wales. Catena.

[bb0125] Collins A.L., Stutter M., Kronvang B. (2014). Mitigating diffuse pollution from agriculture: international approaches and experience. Sci. Total Environ..

[bb0130] Collins A.L., Zhang Y.S., Winter M., Inman A., Jones J.I., Johnes P.J., Cleasby W., Vrain E., Lovett A., Noble L. (2016). Tackling agricultural diffuse pollution: what might uptake of farmer-preferred measures deliver for emissions to water and air. Sci. Total Environ..

[bb0135] Comber S.D.W., Smith S., Daldorph P., Gardner M.J., Constantino C., Ellor B. (2013). Development of a chemical source apportionment decision support framework for catchment management. Environ. Sci. Technol..

[bb0140] Crossman J., Whitehead P.G., Futter M.N., Jin L., Shahgedanova M., Castellazzi M., Wade A.J. (2013). The interactive responses of water quality and hydrology to changes in multiple stressors, and implications for the long-term effective management of phosphorus. Sci. Total Environ..

[bb0145] Cuttle S.P., Newell Price P., Harris D., Chadwick D.R., Shepherd M.A., Anthony S.G., Macleod C.J.A., Haygarth P.M., Chambers B.J. (2016). A method-centric ‘user manual’ for the mitigation of diffuse water pollution from agriculture. Soil Use Manag..

[bb0150] Davison P., Withers P., Lord E., Betson M., Strömqvist J. (2008). PSYCHIC - a process based model of phosphorus and sediment mobilisation and delivery within agricultural catchments. Part 1: model description and parameterisation. J. Hydrol..

[bb0155] Deasy C., Quinton J.N., Silgram M.S., Jackson B., Bailey A.P., Stevens C.J. (2009). Mitigation options for sediment and phosphorus losses from winter-sown arable crops. J. Environ. Qual..

[bb0160] Deb K., Pratap A., Agarwal S., Meyarivan T. (2002). A fast and elitist multi-objective genetic algorithm: NSGA-II. IEEE Trans. Evol. Comput..

[bb0170] Defra (2010). Cost-curves for mitigating multiple water pollutants, ammonia and greenhouse gas emissions on farms – FARMSCOPER decision support tool, user guide and economic analysis for pollution mitigation methods. Defra Project WQ0106.

[bb0175] Defra (2010). Definitions of Terms Used in Farm Business Management.

[bb0180] Defra (2012). Integrating advice on climate change mitigation and adaptation into existing advice packages to achieve multiple wins. Defra Project FF0204.

[bb0185] Defra (2016). The Guide to Cross Compliance in England.

[bb0190] Destandau F., Imfeld G., Rozan A. (2013). Regulation of diffuse pesticide pollution: combining point source reduction and mitigation in stormwater wetland (Rouffach, France). Ecol. Eng..

[bb0195] Doole G.J. (2012). Cost-effective policies for improving water quality by reducing nitrate emissions from diverse dairy farms: an abatement-cost perspective. Agric. Water Manag..

[bb0200] Doole G.J., Marsh D., Ramilan T. (2013). Evaluation of agri-environmental policies for reducing nitrate pollution from New Zealand dairy farms accounting for firm heterogeneity. Land Use Policy.

[bb0205] Duffy A., Moir S., Berwick N., Shabashow J., D'Arcy B., Wade R. (2016). Rural Sustainable Drainage Systems: A Practical Design and Build Guide for Scotland's Farmers and Landowners. http://crew.ac.uk/publications.

[bb0210] Durand P., Breur L., Johnes P.J., van Grinsven H., Butturini A., Billen G., Garnier J., Maberley S., Carvalho L., Reay D., Curtis C., Sutton M.A., Howard C.M., Erisman J.W., Billen G., Bleeker A., Grennfelt P., van Grinsven H., Grizzetti B. (2011). Nitrogen turnover processes and effects in aquatic ecosystems. Chapter 7. European Nitrogen Assessment.

[bb0215] Elofsson K. (2003). Cost-effective reductions of stochastic agricultural loads to the Baltic Sea. Ecol. Econ..

[bb0220] Elofsson K., Folmer H., Gren I.-M. (2003). Management of eutrophicated coastal ecosystems: a synopsis of the literature with emphasis on theory and methodology. Ecol. Econ..

[bb0225] Eory V., Topp C.F.E., Moran D. (2013). Multiple pollutant cost-effectiveness of greenhouse gas mitigation measures in the UK agriculture. Environ. Sci. Pol..

[bb0230] EU COST 869. Mitigation Options for Reducing Nutrient Emissions From agriculture (n.d.).

[bb0235] European Commission, Commission E (2012). Report From the Commission to the European Parliament and the Council on the Implementation of the Water Framework Directive (2000/60/EC) River Basin Management Plans. 3rd Implementation Report.

[bb0240] European Commission (EC) (2000). Directive 2000/60/EC (water framework directive). Off. J. Eur. Communities.

[bb0245] Ferrant S., Durand P., Justes E., Probst J.-L., Sanchez-Perez J.-M. (2013). Simulating the long-term impact of nitrate mitigation scenarios in a pilot study basin. Agric. Water Manag..

[bb0250] Finger R., Calanca P. (2011). Risk management strategies to cope with climate change in grassland production: an illustrative case study for the Swiss plateau. Reg. Environ. Chang..

[bb0255] Fischer P., Pothig R., Venohr M. (2017). The degree of phosphorus saturation of agricultural soils in Germany: current and future risk of diffuse P loss and implications for soil P management in Europe. Sci. Total Environ..

[bb0260] Froschl L., Pierrad R., Schonback W. (2008). Cost-efficient of measures in agriculture to reduce the nitrogen load flowing from the Danube River into the Black Sea: an analysis for Austria, Bulgaria, Hungary and Romania. Ecol. Econ..

[bb0265] Ghebremichael L.T., Veith T.L., Hamlett J.M. (2013). Integrated watershed and farm-scale modelling framework for targeting critical source areas while maintaining farm economic viability. J. Environ. Manag..

[bb0270] Giupponi C., Vladimirova I. (2006). Ag-PIE: GIS-based screening model for assessing agricultural pressures and impacts on water quality on European scale. Sci. Total Environ..

[bb0275] Gooday R.D., Anthony S.G., Chadwick D.R., Newell-Price P., Harris D., Deuthmann D., Fish R., Collins A.L., Winter M. (2014). Modelling the cost-effectiveness of mitigation methods for multiple pollutants at farm scale. Sci. Total Environ..

[bb0280] Gooday R.D., Anthony S.G., Durrant C., Harris D., Lee D., Metcalfe P., Newell-Price P., Turner A. (2015). Farmscoper extension. Final Report for Defra Project SCF0104.

[bb0285] Gren I.-M., Jannke P., Elofsson K. (1997). Cost-effective nutrient reductions to the Baltic Sea. Environ. Resour. Econ..

[bb0290] Gren I.-M., Savchuk O.P., Jansson T. (2013). Cost-effective spatial and dynamic management of a eutrophied Baltic Sea. Mar. Resour. Econ..

[bb0295] Gunningham N., Sinclair D. (2005). Policy instrument choice and diffuse source pollution. J. Environ. Law.

[bb0300] Jacobsen B.H., Anker H.T., Baaner L. (2017). Implementing the water framework directive in Denmark – lessons on agricultural measures from a legal and regulatory perspective. Land Use Policy.

[bb0305] Kramer D.B., Polasky S., Starfield A., Palik B., Westphal L., Snyder S., Jakes P., Hudson R., Gustafson E. (2006). A comparison of alternative strategies for cost-effective water quality management in lakes. Environ. Manag..

[bb0310] Kronvang B., Jeppesen E., Conley D.J., Sondergaard M., Larsen S.E., Ovesen N.B., Carstensen J. (2005). Nutrient pressures and ecological responses to nutrient loading reductions in Danish streams, lakes and coastal waters. J. Hydrol..

[bb0315] Kroon F.J. (2009). Integrated research to improve water quality in the Great Barrier Reef region. Mar. Freshw. Res..

[bb0320] Lacroix A., Beaudoin N., Makowski D. (2005). Agricultural water nonpoint control under uncertainty and climate change. Ecol. Econ..

[bb0325] Lam Q.D., Schmalz B., Fohrer N. (2010). Modelling point and diffuse source pollution of nitrate in a rural lowland catchment using the SWAT model. Agric. Water Manag..

[bb0330] Lam Q.D., Schmalz B., Fohrer N. (2011). The impact of agricultural best management practices on water quality in a North German lowland catchment. Environ. Monit. Assess..

[bb0335] Lescot J.M., Bordenave P., Petit K., Leccia O. (2013). A spatially-distributed costeffectiveness analysis framework for controlling water pollution. Environ. Model. Softw..

[bb0340] McDowell R.W., Nash D. (2013). A review of the cost-effectiveness and suitability of mitigation strategies to prevent phosphorus loss from dairy farms in New Zealand and Australia. J. Environ. Qual..

[bb0345] McGonigle D.F., Harris R.C., McCamphill C., Kirk S., Dils R., Macdonald J., Bailey S. (2012). Towards a more strategic approach to research to support catchment-based policy approaches to mitigate agricultural water pollution: a UK case-study. Environ. Sci. Pol..

[bb0350] McGonigle D.F., Burke S.P., Collins A.L., Gartner R., Haft M.R., Harris R.C., Haygarth P.M., Hedges M.C., Hiscock K.M., Lovett A.A. (2014). Developing Demonstration Test Catchments as a platform for transdisciplinary land management research in England Wales. Environ. Sci.: Processes Impacts.

[bb0355] Meals D.W., Dressing S.A., Davenport T.E. (2010). Lag time in water quality response to best management practices: a review. J. Environ. Qual..

[bb0360] Mockler E.M., Deakin J., Archbold M., Gill L., Daly D., Bruen M. (2017). Sources of nitrogen and phosphorus emissions to Irish rivers and coastal waters: estimates from a nutrient load apportionment framework. Sci. Total Environ..

[bb0365] Moran D., Macleod M., Wall E., Eory V., McVittie A., Barnes A., Rees R., Topp C.F.E., Moxey A. (2010). Marginal abatement–cost curves for UK agricultural greenhouse gas emissions. J. Agric. Econ..

[bb0370] National Audit Office (2010). Environment Agency: Tackling Diffuse Water Pollution in England, Report by the Comptroller and Auditor General.

[bb0375] Newell Price J.P., Harris D., Taylor M., Williams J.R., Anthony S.G., Duethmann D., Gooday R.D., Lord E.I., Chambers B.J., Chadwick D.R., Misselbrook T.H. (2011). Mitigation Methods - User Guide. An Inventory of Mitigation Methods and Guide to Their Effects on Diffuse Water Pollution, Greenhouse Gas Emissions and Ammonia Emissions From Agriculture.

[bb0380] Nix J. (2009). Farm Management Pocket Book.

[bb0385] Ockenden M.C., Deasy C., Quinton J.N., Bailey A.P., Surridge B., Stoate C. (2012). Evaluation of field wetlands for mitigation of diffuse pollution from agriculture: sediment retention cost and effectiveness. Environ. Sci. Pol..

[bb0390] Ockenden M.C., Deasy C., Quinton J.N., Surridge B., Stoate C. (2014). Keeping agricultural soil out of rivers: evidence of sediment and nutrient accumulation within field wetlands in the UK. J. Environ. Manag..

[bb0395] OECD (2012). Water quality and agriculture: meeting the policy challenge. OECD Studies on Water.

[bb0400] Outram F.N., Lloyd C.E.M., Jonczyk J., Benskin C.McW.H., Grant F., Perks M.T., Deasy C., Burke S.P., Collins A.L., Freer J., Haygarth P.M., Hiscock K.M., Johnes P.J., Lovett A.A. (2014). High frequency monitoring of nitrogen and phosphorus response in three rural catchments to the end of the 2011–2012 drought in England. Hydrol. Earth Syst. Sci..

[bb0405] Panagopoulos Y., Makropoulos C., Mimikou M. (2011). Reducing surface water pollution through the assessment of the cost-effectiveness of BMPs at different spatial scales. J. Environ. Manag..

[bb0410] Panagopoulos Y., Makropoulos C., Gkiokas A., Kossida M., Evangelou L., Lourmas G., Michas S., Tsadilas C., Papageorgiou S., Perleros V., Drakopoulou S., Mimikou M. (2014). Assessing the cost-effectiveness of irrigation water management practices in water stressed agricultural catchments: the case of Pinios. Agric. Water Manag..

[bb0415] Patterson J.J., Smith C., Bellamy J. (2013). Understanding enabling capacities for managing the ‘wicked problem’ of nonpoint source water pollution in catchments: a conceptual framework. J. Environ. Manag..

[bb0420] Perez-Martin M.A., Estrela T., del-Amo P. (2016). Measures required to reach the nitrate objectives in groundwater based on a long-term nitrate model for large river basins (Júcar, Spain). Sci. Total Environ..

[bb0425] Perni A., Martinez-Paz J.M. (2013). A participatory approach for selecting cost-effective measures in the WFD context: the Mar Menor (SE Spain). Sci. Total Environ..

[bb0430] Poole A.E., Bradley D., Salazar R., Macdonald D.W. (2013). Optimizing agri-environment schemes to improve river health and conservation value. Agric. Ecosyst. Environ..

[bb0435] Ribaudo M.O., Heimlich R., Claassen R., Peters M. (2001). Least-cost management of nonpoint source pollution: source reductions versus interception strategies for controlling nitrogen loss in the Mississippi Basin. Ecol. Econ..

[bb0440] Rocha J., Roebeling P., Rial-Relvas M.E. (2015). Assessing the impacts of sustainable agricultural practices for water quality improvements in the Vouga catchment (Portugal) using the SWAT model. Sci. Total Environ..

[bb0445] Roebeling P.C., van Grieken M.E., Webster A.J., Biggs J., Thorburn P. (2009). Cost-effective water quality improvement in linked terrestrial and marine ecosystems: a spatial environmental-economic modelling approach. Mar. Freshw. Res..

[bb0450] Roebeling P.C., Rocha J., Nunes J.P., Fidélis T., Alves H., Fonseca S. (2014). Using SWAT to estimate DIN water pollution abatement cost functions in Central Portugal. J. Environ. Qual..

[bb0455] Roebeling P.C., Abrantes N., Ribeiro S., Almeida P. (2016). Estimating cultural benefits from surface water status improvements in freshwater wetland ecosystems. Sci. Total Environ..

[bb0460] Ruitenbeek J., Ridgley M., Dollar S., Huber R. (1999). Optimization of economic policies and investment projects using a fuzzy logic based cost-effectiveness model of coral reef quality: empirical results for Montego Bay, Jamaica. Coral Reefs.

[bb0465] Schou J.S., Skop E., Jensen J.D. (2000). Integrated agri-environmental modelling: a cost effectiveness analysis of two nitrogen tax instruments in the Vejle Fjord watershed, Denmark. J. Environ. Manag..

[bb0470] Schoumans O.F., Chardon W.J., Bechmann M., Gascuel-Odoux C., Hofman G., Kronvang B., Litaor M.I., Lo Porto A., Newell-Price P., Rubaek G. (2011). Mitigation Options for Reducing Nutrient Emissions From Agriculture: A Study Amongst European Member States of COST Action 869. Alterra Report 2141, Alterra, Wageningen, The Netherlands.

[bb0475] Semaan J., Flichman G., Scardigno, Steduto P. (2007). Analysis of nitrate pollution control policies in the irrigated agriculture of the Apulia Region (Southern Italy): a bio-economic modelling approach. Agric. Syst..

[bb0480] Shang X., Wang X., Zhang D., Chen W., Chen X., Kong H. (2012). An improved SWAT-based computational framework for identifying critical source areas for agricultural pollution at the lake basin scale. Ecol. Model..

[bb0485] Stromqvist J., Collins A.L., Davison P.S., Lord E.I. (2008). PSYCHIC – a process-based model of phosphorus and sediment transfers within agricultural catchments. Part 2. A preliminary evaluation. J. Hydrol..

[bb0490] Teshager A.D., Gassman P.W., Secchi S., Schoof J.T. (2017). Simulation of targeted mitigation-strategies to reduce nitrate and sediment hotspots in agricultural watershed. Sci. Total Environ..

[bb0495] Thorburn P. (2013). Catchments to reef continuum: minimising impacts of agriculture on the Great Barrier Reef. Agric. Ecosyst. Environ..

[bb0500] Trepel M. (2010). Assessing the cost-effectiveness of the water purification function of wetlands for environmental planning. Ecol. Complex..

[bb0505] van Grieken M.E., Thomas C.R., Roebeling P.C., Thorburn P.J. (2013). Integrating economic drivers of social change into agricultural water quality improvement strategies. Agric. Ecosyst. Environ..

[bb0510] Veith T., Wolfe M., Heatwole C. (2003). Optimization procedure for cost effective BMP placement at a watershed scale. J. Am. Water Resour. Assoc..

[bb0515] Vinten A.J.A., Martin-Ortega J., Glenk K., Booth P., Balana B.B., MacLeod M., Lago M., Moran D., Jones M. (2012). Application of the WFD cost proportionality principle to diffuse pollution mitigation: a case study for Scottish Lochs. J. Environ. Manag..

[bb0520] Vinten A.J.A., Sample J., Ibiyemi A., Abdul-Salam Y., Stutter M. (2017). A tool for cost-effectiveness analysis of field scale sediment-bound phosphorus mitigation measures and application to analysis of spatial and temporal targeting in the Lunan Water catchment, Scotland. Sci. Total Environ..

[bb0525] Wang L., Stuart M.E., Lewis M.A., Ward R.S., Skirvin D., Naden P.S., Collins A.L., Ascott M.J. (2016). The changing trend in nitrate concentrations in major aquifers due to historical nitrate loading from agricultural land across England and Wales from 1925 to 2150. Sci. Total Environ..

[bb0530] WATECO (2003). Common Implementation Strategy for the Water Framework Directive (2000/60/EC). Guidance Document No. 1. Economics and Environment — the Implementation Challenge of the WFD European Commission, Luxembourg.

[bb0535] Whitehead P.G., Wade A.J., Butterfield D. (2009). Potential impacts of climate change on water quality and ecology in six UK rivers. Hydrol. Res..

[bb0540] Wilkinson M.E., Quinn P.F., Barber N.J., Jonczyk J. (2014). A framework for managing runoff and pollution in the rural landscape using a Catchment Systems Engineering approach. Sci. Total Environ..

[bb0545] Wright S.A.L., Fritsch O. (2011). Operationalising active involvement in the EU Water Framework Directive: why, when and how?. Ecol. Econ..

[bb0550] Wright S.A.L., Jacobsen B.H. (2011). Participation in the implementation of the Water Framework Directive in Denmark: the prospects for active involvement. Water Policy.

[bb0555] Yang W., Rousseau A.N., Boxall P. (2007). An integrated economic-hydrologic modeling framework for the watershed evaluation of beneficial management practices. J. Soil Water Conserv..

[bb0560] Zessner M., Schonhart M., Parajka J., Trautvetter H., Mitter H., Kirchner M., Hepp G., Blaschke A.P., Strenn B., Schmid E. (2017). A novel integrated modelling framework to assess the impacts of climate and socio-economic drivers on land use and water quality. Sci. Total Environ..

[bb0565] Zhang Y., Collins A.L., Gooday R.D. (2012). Application of the FARMSCOPER tool for assessing agricultural diffuse pollution mitigation methods across the Hampshire Avon Demonstration Test Catchment, UK. Environ. Sci. Pol..

[bb0570] Zhang Y., Collins A.L., Murdoch N., Lee D., Naden P.S. (2014). Cross sector contributions to river pollution in England and Wales: updating waterbody scale information to support policy delivery for the Water Framework Directive. Environ. Sci. Pol..

[bb0575] Zhang Y., Collins A.L., Johnes P.J., Jones J.I. (2017). Projected impacts of increased uptake of source control mitigation measures on agricultural diffuse pollution emission to water and air. Land Use Policy.

[bb0580] Zhang Y., Collins A.L., Jones J.I., Johnes P.J., Inman A., Freer J.E. (2017). The potential benefits of on-farm mitigation scenarios for reducing multiple pollutant loadings in prioritised agri-environment areas across England. Environ. Sci. Pol..

