# Supplementary material for: Assessing the potential impacts of a revised set of on-farm nutrient and sediment ‘basic’ control measures for reducing agricultural diffuse pollution across England
Source: Sci Total Environ. 2018 Apr 15;621:1499–511. doi: 10.1016/j.scitotenv.2017.10.078 (PMC5805857; doi:10.1016/j.scitotenv.2017.10.078)

**Supplementary Information**

**Soil types**

Table S1: The correspondence between HOST classes and FARMSCOPER soil categories.

| HOST class | Soil group | HOST class | Soil group |
| --- | --- | --- | --- |
| 1 | Free draining | 15 | Free draining |
| 2 | Free draining | 16 | Free draining |
| 3 | Free draining | 17 | Free draining |
| 4 | Free draining | 18 | Drained for arable |
| 5 | Free draining | 19 | Drained for arable |
| 6 | Free draining | 20 | Drained for arable |
| 7 | Free draining | 21 | Drained for arable |
| 8 | Free draining | 22 | Drained for arable |
| 9 | Drained for arable | 23 | Drained for both arable and grass |
| 10 | Drained for arable | 24 | Drained for both arable and grass |
| 11 | Free draining | 25 | Drained for both arable and grass |
| 12 | Free draining | 26 | Free draining |
| 13 | Free draining | 27 | Free draining |
| 14 | Drained for arable | 28 | Free draining |

**Farm types**

FARMSCOPER has 15 built-in farms listed in Table S1. Based on national summary statistics, each has its own typical cropping area, livestock counts and associated fertiliser application and manure management rates / practices. The former is based on the survey of British Practice of Fertiliser Application (BPFP, more information at https://www.gov.uk/government/collections/fertiliser-usage) and the latter is derived from the Farm Practice Survey (FPS, more information at https://www.gov.uk/government/collections/farm-practices-survey). For the modelling exercise reported in the paper to which this supplementary document provides support, 2010 June Agriculture Survey (JAS) data were used to map farm types and to populate the corresponding cropping areas and livestock numbers to create customised farms for modelling scenarios. Nine default farm types were used in the generation of FARMSCOPER model farms for the modelling exercise reported here and those farm systems are highlighted in bold in the table below with corresponding Defra Robust Farm Types (RFTs) clarified in the brackets.

Table S2: Relationship between FARMSCOPER model farms and the RFT typology.

| **Dairy (Dairy)** | **Horticulture (Horticulture)** |
| --- | --- |
| **LFA grazing (Upland Grazing Livestock)** | Mixed combinable with poultry manure |
| **Lowland grazing (Lowland Grazing Livestock)** | Roots & combinable |
| **Mixed livestock (Mixed)** | Roots & combinable with poultry manure |
| **Indoor pig (Specialist Pigs)** | Outdoor pig |
| **Mixed combinable (Cereals)** | Indoor pigs plus mix combinable |
| **Specialist poultry (Specialist Poultry)** | Indoor pigs plus winter combinable |
| **Winter combinable (General Cropping)** |  |

**Cost calculations**

The cost of agricultural diffuse pollution mitigation was calculated as the sum of the amortised annual farm costs of the individual mitigation methods selected by an optimisation scenario. Costs were calculated net of any prior method implementation. The costs represented the cost to the farm sector only, and excluded any costs to government in administrating any scheme that supported or enforced method implementation. The implementation of a pollutant mitigation method may alter farm finances by changing the variable costs and gross margin of a crop or stock enterprise; by changing the fixed costs or overheads associated with labour and

Machinery, or; by requiring a capital investment. Mitigation methods may give rise to costs in more than one category. Mitigation method costs in the literature are normally reported as a net cost for a specific farm system. To enable scaling of the costs and application to a range of farm types and sizes, the available cost data are re-expressed as unit costs in one of three ways:

• Excreta unit cost

The annual mitigation method cost is expressed per cubic metre of all livestock excreta produced on a farm. The assumption is that the cost represents farm inputs that are directly in proportion to the numbers of animals, and hence the total quantity of excreta produced on the farm. For example, dietary supplements are in proportion to the animal diet and therefore excreta production, and; the roofing of concrete yards will be in proportion to the yard size that is also in proportion to animal numbers or excreta production. In effect, excreta production is used as a form of livestock unit.

• Manure unit cost

The annual mitigation method cost is expressed per cubic metre of managed slurry or farm yard manure on a farm. The assumption is that the cost represents additional handling and storage costs that are in proportion to the quantity of manure. For example, restrictions on the placement of manure next to watercourses require additional planning time, and; restrictions on timing of manure application may require additional storage facilities.

• Area unit cost

The annual mitigation method cost is expressed per hectare of arable, grass or rough grazing on the farm. The assumption is that the cost represents income foregone or labour that is in proportion to the land area. For example, riparian buffer zones require land to be taken out of production, and; the cultivation of compacted soils requires an extra tillage operation.

**Control measure efficacy classes and corresponding uncertainty ranges**

Table S3: Average efficacy classes and corresponding uncertainty ranges.

| Efficacy class | Average efficacy | Uncertainty range | Pollutant reduction |
| --- | --- | --- | --- |
| A | - | - | None |
| B | 2 | 0-10 | Very low |
| C | 10 | 2- 25 | Low |
| D | 25 | 10-50 | Moderate |
| E | 50 | 25-80 | High |
| F | 80 | 50-95 | Very high |
| G | 100 | 0 | Total |

**Rainfall and soil combinations**

Table S4: Summary of the soil group, rainfall band and RFT combinations in each DTC.

| DTC | Number of soil groups | Number of rainfall bands | Selected RFTs |
| --- | --- | --- | --- |
|  |  |  |  |
| Eden | 2 | 4 | Dairy, LFA^1^ Grazing, Lowland Grazing Livestock, Mixed |
| Wensum | 3 | 2 | Cereal, General Cropping, Dairy, Lowland Grazing Livestock, Specialist Pigs |
| Avon | 3 | 2 | Cereal, Dairy, Lowland Grazing Livestock, Mixed |
| Tamar | 3 | 3 | Cereal, Dairy, Lowland Grazing Livestock, Mixed, LFA^1^ Grazing |

^1^LFA – less favoured area

**‘First-filter’ measures (n=90)**

Table S5: Potential (n = 90) measures for nutrient and sediment control identified by the preliminary screening exercise (X = applicable; - = not applicable; (-) = no robust evidence available; (🡡) = risk of increasing pollutant emission; CSF = Catchment Sensitive Farming initiative; NVZ = Nitrate Vulnerable Zone; SSAFO = Silage, Slurry and Agricultural Fuel Oil regulations). ^1^ indicates measure failed to gain industry support at the stakeholder workshop.

| Measure | Applicability to agricultural nutrient and sediment pollution control | | |  |
| --- | --- | --- | --- | --- |
| Farmyard surface and drainage infrastructure and management options | Phosphorus | Nitrate | Sediment | Policy mechanism |
| Livestock feed or bedding materials are stored away from areas at risk of flooding | X | - | - | Voluntary |
| Livestock feeds are stored under cover and any effluent is contained | X | - | - | Voluntary |
| Fertiliser is stored securely and under cover (away from combustible material – straw, Diesel etc)^1^ | X | X | - | Voluntary |
| Used fertiliser bags, pesticide containers are secure & under cover | X | X | - | Agricultural waste regulations |
| Ensure that your fertiliser store is secure and located more than 10m away from a watercourse and/or drain^1^ | X | X | - | Voluntary |
| Septic tanks are managed or upgraded and fit for purpose | X | X | - | Voluntary |
| Existing yard drainage systems are assessed and any misconnections repaired | X | X | - | CSF capital grant |
| Repair or install roof gutters and down pipes to divert rainfall inputs to clean water drainage and/or subsequent re-use | X | X | - | CSF capital grant |
| Contaminated yard water is diverted to secure storage for land spreading | X | X | - | CSF capital grant |
| Create reed beds as final treatment system (e.g. for yard runoff) ^1^ | X | X | - | Voluntary |
| Establish and maintain constructed wetlands (e.g. for yard runoff) ^1^ | X | X | - | Voluntary |
| Make silage to minimise effluent | X | X | - | Voluntary |
| Silage effluent storage in place to cover periods of inappropriate weather | X | X | - | SSAFO |
| Silage effluent is contained, collected and stored | X | X | - | SSAFO |
| Minimum slurry storage calculation for 1 in 5 year rainfall over the 4 month winter (Nov-Feb) period | X | X | - | SSAFO |
| Slurry storage in place for 4 month period for inappropriate weather | X | X | - | SSAFO |
| Increase the capacity of farm manure (slurry) stores | X | X | - | NVZ rule |
| Transport manure to neighbouring farms | X | X | - | NVZ rule |
| Farm track management | X | X | X | CSF capital grant |
| Construct road bunds / cross drains to intercept runoff channelled on tracks^1^ | X | X | X | CSF capital grant |
| Construct Swales/Soak aways (e.g. for track runoff) ^1^ | X | X | X | Voluntary |
| Wetland creation (e.g. for track runoff) ^1^ | X | X | X | Voluntary |
| Dredge/de-silt field ditches | X (🡡) | X (🡡) | X (🡡) | Voluntary |
|  | | | | |
| Field/soil/land management | Phosphorus | Nitrate | Sediment | Policy mechanism |
| Avoid irrigating when it rains^1^ | X | - | X | Voluntary |
| Avoid irrigating when it is windy^1^ | X | - | X | Voluntary |
| Ensure irrigation is applied to prevent capping & excess runoff^1^ | X | - | X | Voluntary |
| Plan irrigation to maximise nutrient uptake | - | X | - | Voluntary |
| Site temporary solid manure heaps away from watercourses | X | X | - | NVZ rule |
| Do not locate temporary organic manure field heaps on arable land in the same place as an earlier one constructed within the last two years | (-) | X | (-) | NVZ – practical implications |
| Do not locate temporary organic manure field heaps on grassland in the same place as an earlier one constructed within the last two years | (-) | X | (-) | NVZ – practical implications |
| Do not locate organic manure field heaps in any single position for more than 12 consecutive months or within 10 m of an effective drain | X | X | - | NVZ rule |
| Complete a soil risk record based on field soil type, degradation threats and impact of wind and runoff | X | - | X | Cross Compliance |
| Assess risk of soil erosion by water and wind | X | - | X | Cross Compliance |
| Rotate stock to avoid nutrient build-up or increase rotation^1^ | X | X | X | Voluntary |
| Rotate stock more frequently to reduce risk of poaching | X | X | X | Voluntary |
| Reduce field stocking rates when soils are wet and susceptible to poaching or compaction (or at risk of flooding) ^1^ | X | X | X | Voluntary |
| Remove (house) livestock from fields during 'at-risk' seasons^1^ | X | X | X | Voluntary |
| Winter removal of livestock in fields with watercourses^1^ | X | X | X | Higher Level agri-environment scheme |
| Move (field) livestock feeders at frequent intervals to minimise poaching | X | X | X | Entry Level agri-environment scheme – practical implications |
| Minimise poaching from supplementary livestock feeding | X | X | X | Voluntary |
| Ensure suitability of land for outdoor livestock production | X | - | X | Voluntary |
| No overgrazing of natural or semi- natural grassland | X | X | X | Habitat Directive and Cross Compliance |
| Heather and grass burning guidelines followed | X | - | X | Cross Compliance |
| Bracken is controlled to prevent erosion^1^ | X | - | X | Voluntary |
| Avoid working wet soils | X | - | X | Voluntary |
| Time cultivations carefully with respect to prevailing weather and soil conditions | X | - | X | Voluntary |
| Avoid erosion prone crops e.g. potatoes on erosion prone soils e.g. sands^1^ | X | - | X | Voluntary |
| Restrict use of high risk crops to low risk fields (e.g.: potatoes, root crops, bulbs & vegetables) ^1^ | X | - | X | Voluntary |
| Site and manage plastic mulch and polytunnels to minimise runoff | X | - | X | Voluntary |
| Incorporate straw to protect emerging seedlings on risky soils from wind erosion^1^ | X | - | X | Voluntary |
| Use correctly inflated low ground pressures tyres/wheels on machinery^1^ | X | - | X | Voluntary |
| Keep a record of activities on waterlogged soil^1^ | - | - | - | Cross Compliance |
| No mechanical operations take place on waterlogged soils | X | - | X | Voluntary |
| Take action to remediate any damage caused by accessing waterlogged land | X | - | X | Cross Compliance |
| Before establishing the following crop, remove compaction by loosening the topsoil or sub-soiling where necessary. | X | - | X | Voluntary |
| Post harvest management - land over winter is left with a rough surface following operations | X | X | X | Cross Compliance |
| Post harvest management - stubbles are left over winter | X | X | X | Cross Compliance |
| Post harvest management -Sow autumn crop within 10 days of preparing the seedbed | X | X | X | Cross Compliance |
| Establish winter cover crops to reduce nitrate leaching *on land where soil would normally be left bare during winter) ^1^ | X | - | X | Entry Level agri-environment scheme – practical implications |
| Manage over-winter tramlines to minimise soil erosion and runoff^1^ | X | - | X | Voluntary |
| Where soil organic matter content is low improve through inputs such as organic matter from sources such as livestock manure, compost, biosolids etc. | X (🡡) | (🡡) | X | Voluntary |
| Do not destroy hedges | X | X | X | Hedgerow regulations |
| Maintain hedges (height, cutting regime & timing) | X | X | X | Cross Compliance and Voluntary |
|  | | | | |
| Nutrient/manure management planning and application | Phosphorus | Nitrate | Sediment | Policy mechanism |
| Soil test every 5 years, N, P, K, pH, & organic matter content | X | X | - | Voluntary |
| Use a fertiliser recommendation system | X | X | - | Voluntary |
| Produce a nutrient management plan each year for nitrogen for organic manures and manufactured fertiliser | - | X | - | Voluntary |
| Use fertiliser recommendation system for planning phosphate additions | X | - | - | Voluntary |
| Do not apply any P (organic or manufactured) to high P index soils on fields at risk of soil erosion and runoff (sloping land adjacent to a watercourse or fields susceptible to flooding) | X | - | - | Voluntary |
| Do not apply manufactured P fertiliser to high P index soils | X | - | - | Voluntary |
| Calibrate fertiliser spreader^1^ | - | X | - | NVZ rule |
| Use fertiliser spreading equipment with a low spreading trajectory, that is, below 4 metres from the ground | - | X | - | Voluntary |
| Do not apply manufactured fertiliser to high-risk areas | X | X | - | NVZ rule |
| Avoid spreading manufactured fertiliser to fields at high-risk times when soil is waterlogged, flooded, frozen hard, or snow covered. | X | X | - | NVZ rule |
| Spread organic manures to land in compliance with a Manure Management Plan | X | X | - | NVZ rules (risk map) |
| Restrict total nitrogen application to field from organic manures (250kg N/ha) | X | X | - | NVZ rule |
| Restrict total Nitrogen field input from organic manure | X | X | - | NVZ rule |
| Prohibit application of high readily available N organic manures within closed periods | X | X | - | NVZ rule |
| Do not apply organic manures within 10 m of a watercourse, 50 m spring, well or borehole | X | X | - | Cross Compliance |
| Do not apply organic manures to high-risk areas | X | X | - | NVZ rule |
| Do not spread organic manure to fields at high-risk times when the soil is waterlogged; or flooded; or frozen hard; or snow covered. | X | X | - | NVZ rule |
| Do not spread slurry or poultry manure at high-risk times when the soil is waterlogged; or flooded; or frozen hard; or snow covered | X | X | - | NVZ rule |
| Do not use high trajectory (over 4m) spreading equipment | X | X | - | NVZ rule |
| Rapid incorporation of FYM and slurry into the soil (24 hours at the latest, preferably within 6 hours of application) | X | (🡡) | - | NVZ rule |
|  | | | | |
| Riparian management | Phosphorus | Nitrate | Sediment | Policy mechanism |
| Leave a 1m (from the top of the ditch bank) uncultivated buffer next to watercourses | X | X | X | Cross Compliance |
| Do not spread manufactured fertiliser within 2 m of a watercourse | - | X | - | Cross Compliance |
| Locate out-wintered stock and sacrifice areas away from watercourses^1^ | X | X | X | Voluntary |
| Reduce field stocking rates on fields adjacent to watercourses when soils are wet and susceptible to poaching or compaction (or at risk of flooding) | X | X | X | Voluntary |
| Site livestock feeders away from watercourses | X | X | X | Voluntary |
| Site stock water troughs or drinkers away from rivers^1^ | X | X | X | Voluntary |
| Grass or woodland buffer strips on sloping land adjacent to watercourses to intercept runoff and filter sediment^1^ | X | X | X | Voluntary |

Figure S1. The Demonstration Test Catchments (moving clockwise from top left – Eden, Wensum, Hampshire Avon, Tamar).


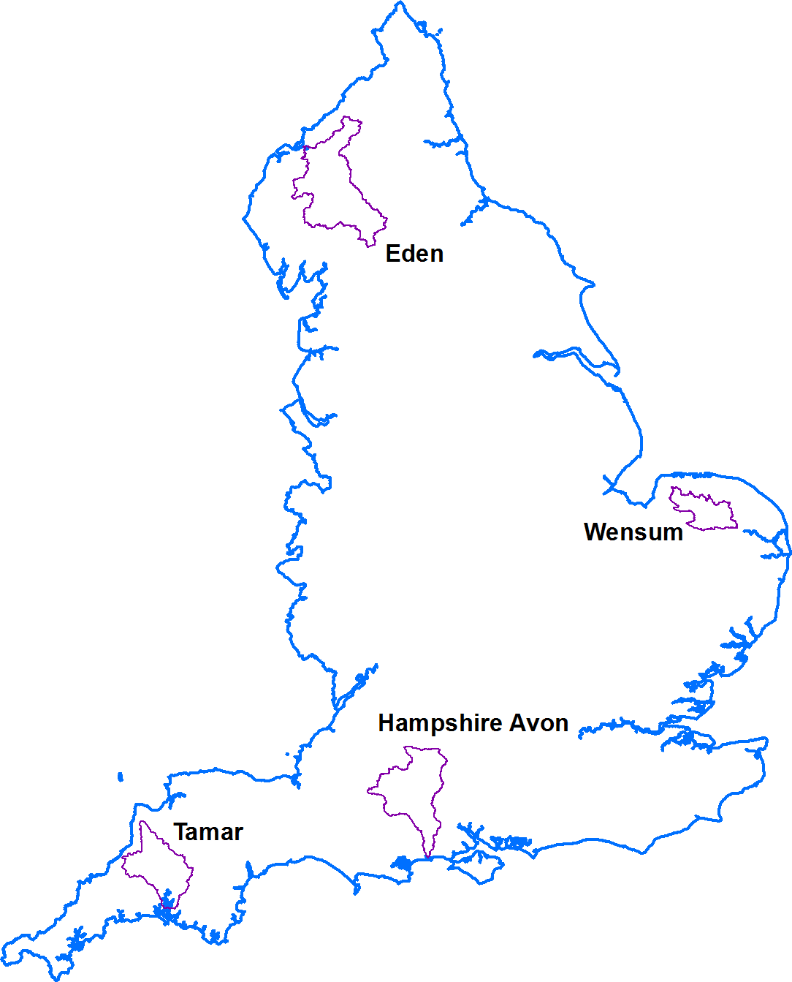


Figure S2. The WMC boundaries across England.


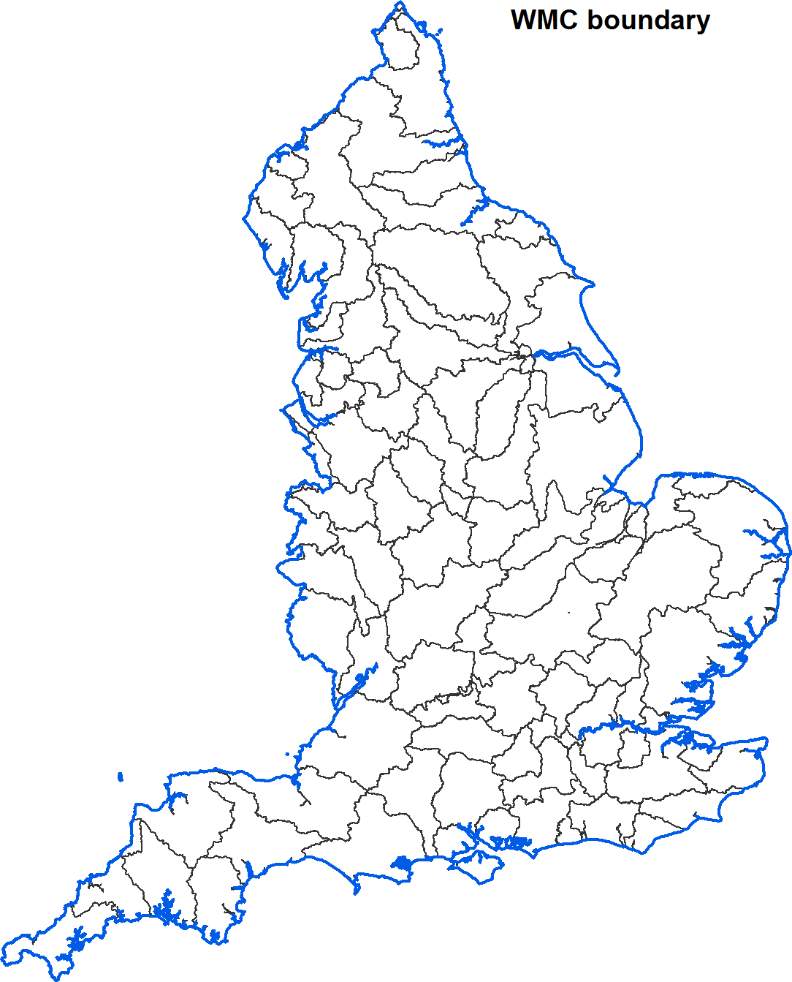


Figure S3. Contribution from agriculture to the total pollutant load delivered to watercourses for each WMC for a) nitrate, b) phosphorus, and c) sediment.


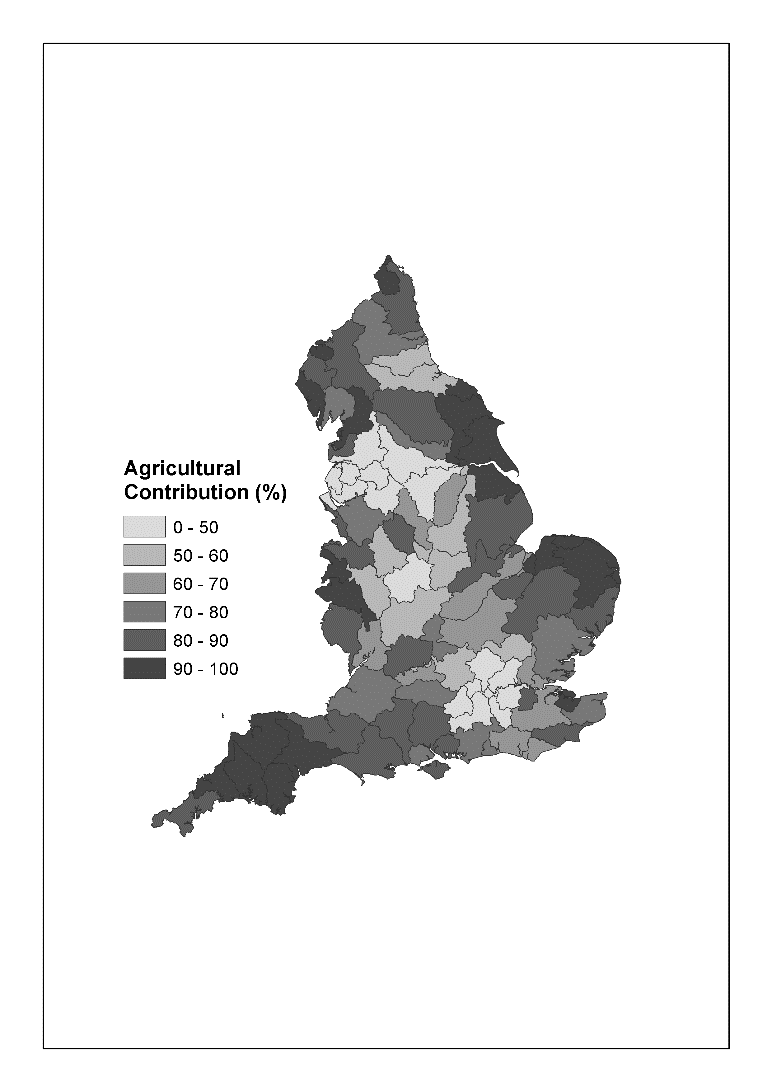


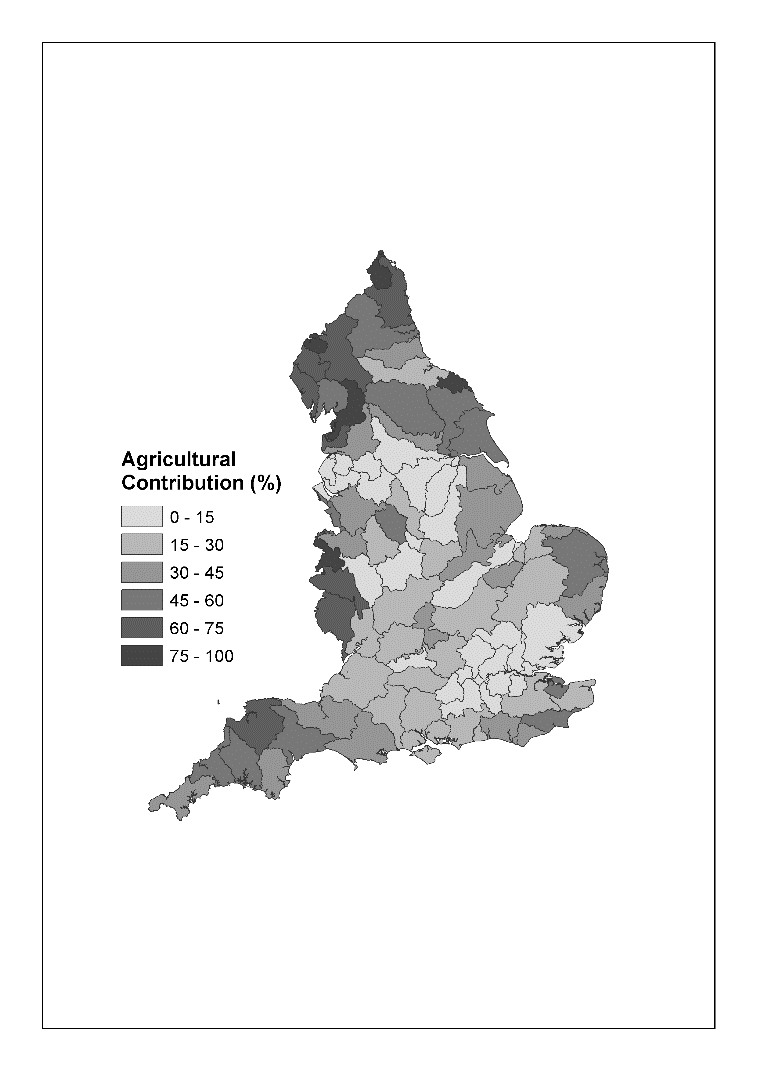


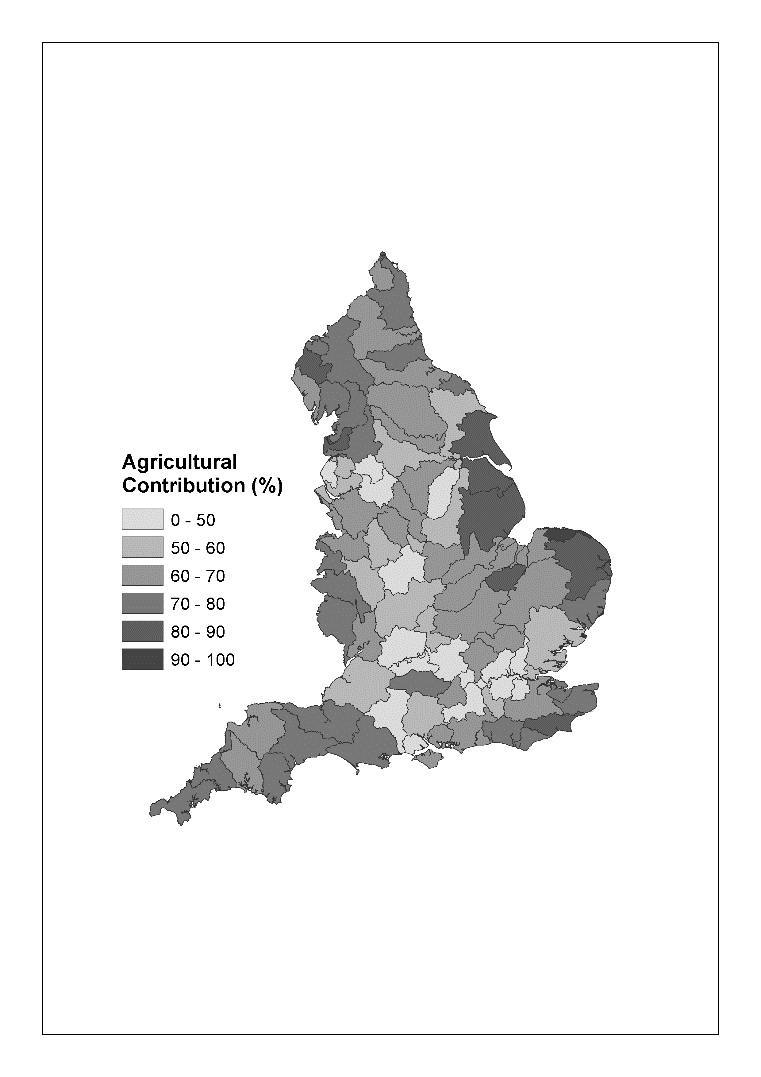


Figure S4. Impact of the suite of ‘basic’ measures on pollution loads delivered to watercourses for each WMC for a) nitrate b) phosphorus, and c) sediment, accounting for the contributions to the total load from all sectors.


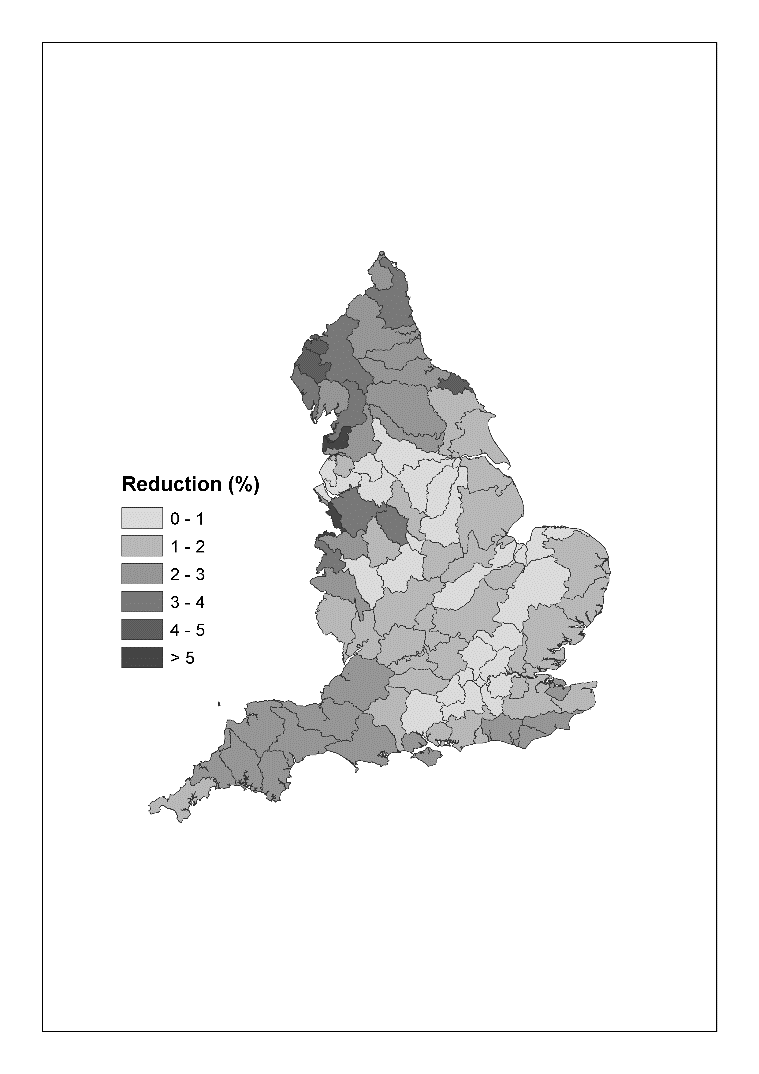


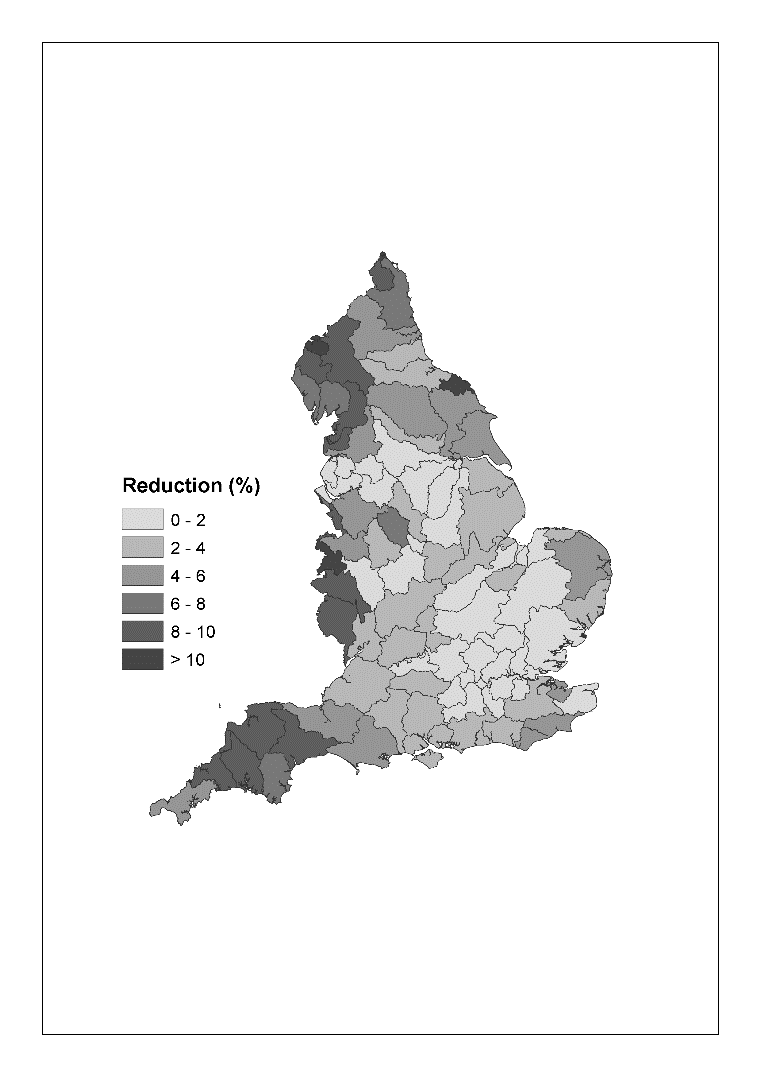


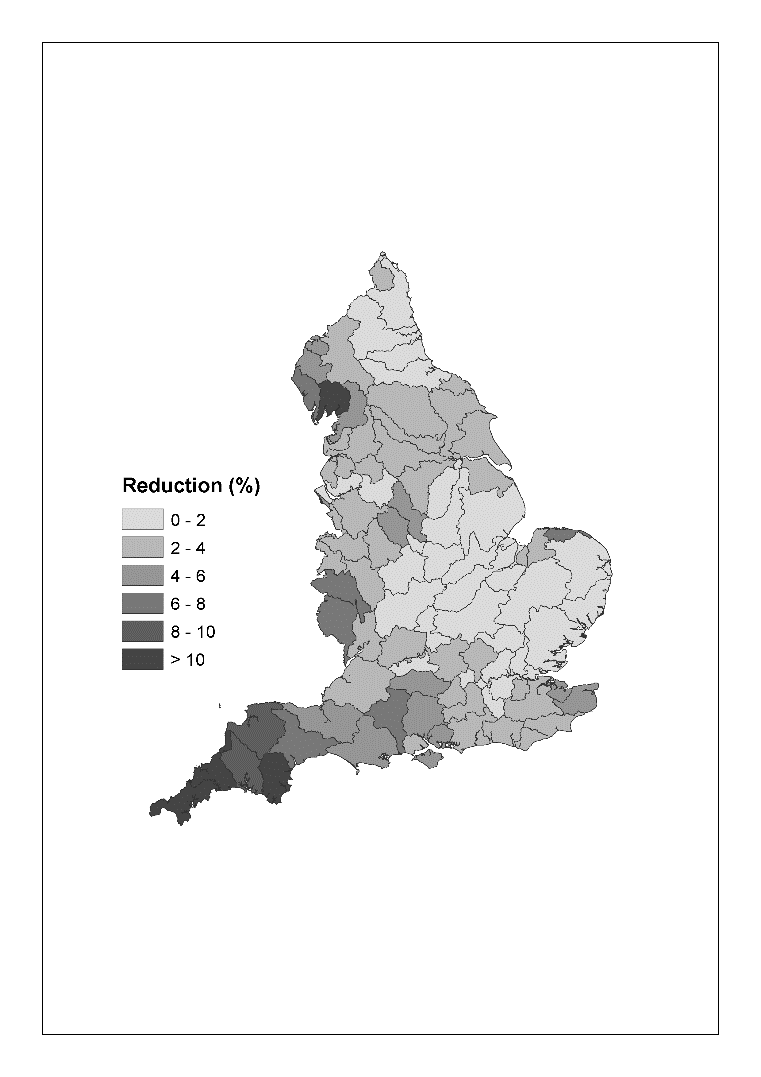

Supplement: Supplementary file 1 — Supplementary material [file mmc1.docx]
